# Similarity and difference in tumor-infiltrating lymphocytes in original tumor tissues and those of *in vitro* expanded populations in head and neck cancer

**DOI:** 10.18632/oncotarget.23454

**Published:** 2017-12-19

**Authors:** Lili Ren, Tatsuo Matsuda, Boya Deng, Kazuma Kiyotani, Taigo Kato, Jae-Hyun Park, Tanguy Y. Seiwert, Everett E. Vokes, Nishant Agrawal, Yusuke Nakamura

**Affiliations:** ^1^ Department of Medicine, The University of Chicago, Chicago, IL, USA; ^2^ Department of Surgery, The University of Chicago, Chicago, IL, USA; ^3^ Cancer Precision Medicine Center, Japanese Foundation for Cancer Research, Tokyo, Japan; ^4^ Cytotherapy Laboratory, Shenzhen People’s Hospital (The second Clinical Medical College of Jinan University), Shenzhen, China

**Keywords:** mismatch repair, non-synonymous mutation, squamous cell carcinoma of head and neck cancer, T cell receptor, tumor-infiltrating lymphocytes

## Abstract

Though adoptive tumor-infiltrating lymphocyte (TIL) therapy has been explored in clinical trials for many years, there is little information for the clonotype composition between TILs in original tumor tissues and TILs that were *in vitro* expanded and infused to cancer patients. To investigate the similarity/difference in TILs in original tumor tissues and those of *in vitro* expanded populations in squamous cell carcinoma of head and neck (SCCHN) as well as their correlation with somatic mutations in cancer cells, we performed whole exome analysis, expression profile analysis of immune-related genes, and T cell receptor (TCR) analysis of original TILs and *in vitro* expanded TILs in 8 surgically-resected HPV-negative fresh tumors with SCCHN. We found an unusually high number of non-synonymous somatic mutations (4290, 1779 and 901 mutations) in three SCCHN tumors, in which we identified mutations in mismatch repair genes, *MSH2* or *MSH4*, or a DNA polymerase gene, *POLE*. Interestingly, dominant TCR clonotypes of expanded CD8^+^ TILs derived from these three tumors revealed high similarity to those in original tumors while for remaining tumors with the lower mutational load, we found that T cell clonotypes between TILs in original tumor tissues and those expanded *in vitro* were almost entirely different. Our findings might provide clinically useful information for identification of tumor-antigen-specific T cell clones that may lead to further improvement of adoptive TIL therapy for SCCHN patients.

## INTRODUCTION

Squamous cell carcinoma of head and neck (SCCHN) is the sixth common cancer worldwide with more than 550,000 new cases and 380,000 deaths annually [1]. Even with improvements in curative intent therapy such as chemoradiotherapy and multimodality treatments, the 5-year disease-specific survival is approximately 50-60%. Hence, novel treatment modalities are urgently needed [2]. Accumulated evidences have shown that certain levels of anti-tumor T cells are present in the tumor microenvironment of a subset of patients who may benefit from anti-immune checkpoint antibody therapy [3–5] or from adoptive tumor-infiltrating lymphocyte (TIL) therapy [6–8]. However, we still do not have sufficient information for TILs function and representativeness in TIL products as well as biomarkers that may help predict the clinical outcome.

Recently, Le *et al.* reported that mismatch repair (MMR) deficiency in tumors regardless of anatomic sites associates with clinical response to the programmed death-1 (PD-1) blockade and that a large number of neoantigens generated by the MMR deficiency in cancer cells increase the sensitivity to the immune checkpoint blockades [9]. Other reports have also indicated that MMR deficiency is a predictive biomarker for immunotherapy response based on clinical trial data from patients with colorectal cancer, cholangiocarcinoma, endometrial cancer and gastric cancer [10–12].

Clinical response to immune checkpoint blockades is related to the function of TILs targeting tumor-specific antigens [13]. Hence, in this study we aimed to characterize the somatic mutational profiles, expression profiles of immune-related genes, and T cell clonality in SCCHN tumors, as well as to compare T cell receptor (TCR) profiles of TILs in original tumor tissues with those of *in vitro* expanded T cell populations that are often used for adoptive T cell therapy.

We here report that TCR clonotypes in original TILs and expanded TILs differ markedly, except in three tumors, which had very high mutational burden due to the MMR deficiency or a DNA polymerase mutation. We suspect that tumors with higher mutations may have a higher number of immune active neoantigens and higher levels of activated T cells, which are likely to predominantly expand in *in vitro* culture. Our data may be helpful in the future for selection of patients for adoptive TIL treatment of SCCHN cancers and might be applicable for identification of T cells recognizing tumor-specific antigens.

## RESULTS

### Exome sequencing of SCCHN tumors

We obtained 8 surgically-resected HPV-negative fresh tumors as well as blood samples/adjacent normal tissues from 8 patients with SCCHN. The clinical characteristics of these patients are summarized in Table 1. We performed whole exome sequencing using DNAs isolated from blood/adjacent normal tissue and tumor DNAs. We obtained an average sequencing depth of 74.2X per base and identified a total of 7,207 non-synonymous mutations in these 8 tumors (11 to 4,290 mutations per sample).

**Table 1:** Clinicopathological characteristics of 8 patients with SCCHN

Patients	Age	Sex	Smoking status	Stage	TNM	HPV status	Anatomic site	Expanded TILs
B1	66	male	>10py	IVA	T2N2b-c	Neg	left base of tongue	No
B2	89	male	>10py	II	T2Nx	Neg	left ear	Yes
B3	84	male	>10py	I	T1Nx	Neg	right parotid gland	Yes
B4	44	male	0-10py	II	T2N0	Neg	left posterior tongue	Yes
B5	84	male	>10py	II	T2Nx	Neg	skin of face	Yes
B6	59	male	0-10py	IVA	T2N2b-c	Neg	right tonsil	Yes
B7	63	male	>10py	II	T2N0	Neg	left tonsil	Yes
B8	77	male	0-10py	IVA	T4N0	Neg	right parotid gland	Yes

py: packs/year.

In three patients, B2, B5 and B8, a very high number of non-synonymous mutations were identified (1,779, 4,290, and 901 mutations, respectively). Due to the unusually high mutational loads in the three cases, we checked whole exome sequencing data and identified possible causes of these high numbers of mutations in genes involved in the mismatch repair process (DNA mismatch repair proteins, *MSH2* in B2 and *MSH4* in B8) and the DNA replication process (DNA polymerase epsilon-*POLE* in B5); we found non-synonymous mutations causing amino acid substitutions of G338R in *MSH2* (B2), H475Y in *POLE* (B5), and P14S in *MSH4* (B8). Mutations in other genes that might also involve in the DNA damage repair process are summarized in Supplementary Table 1.

### Immune microenvironment in SCCHN tumors with high number of mutations

**Figure 1:**
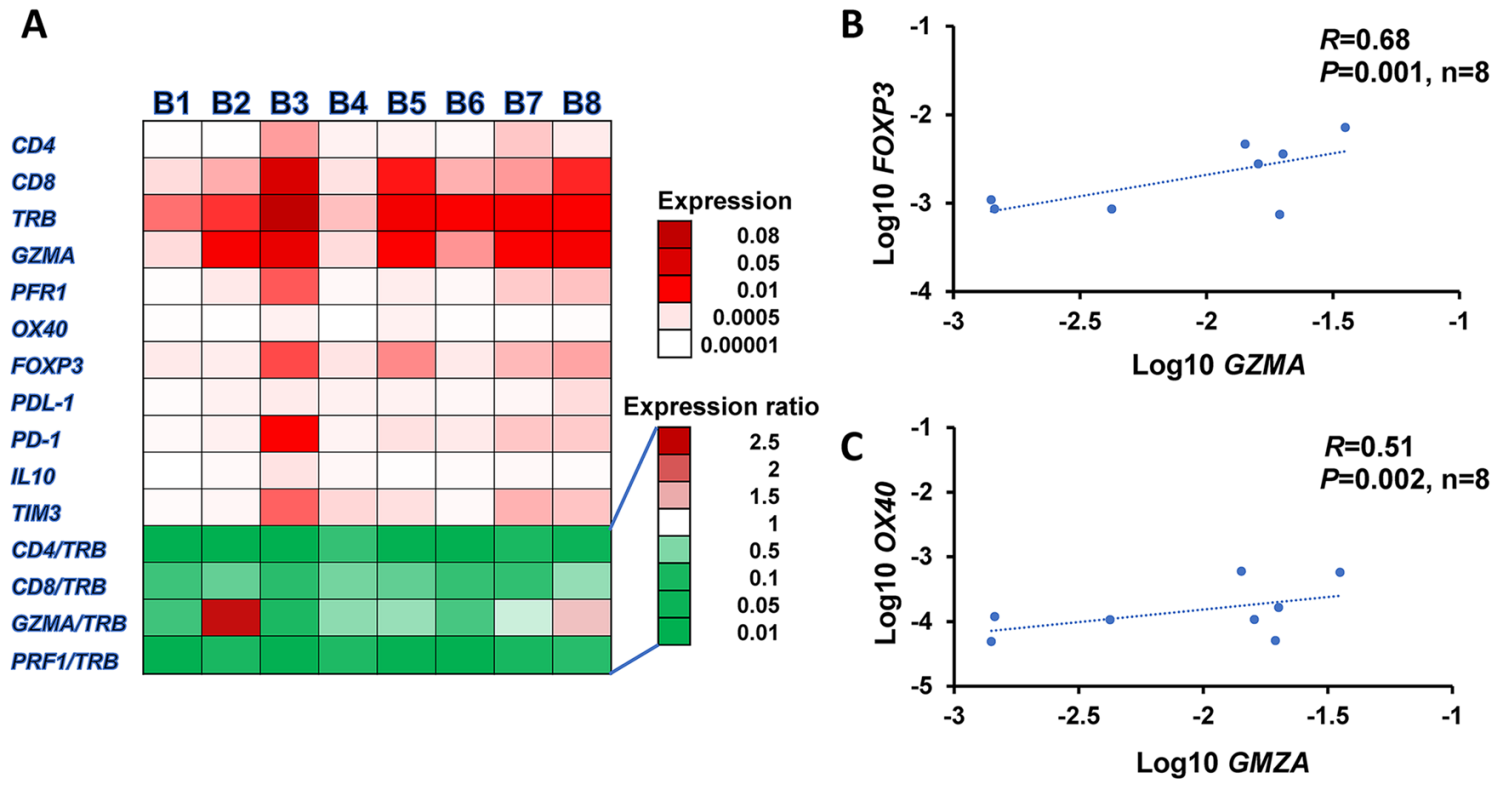
Expression profiles of immune-related genes in tumor microenvironment **(A)** Heatmap of mRNA expression levels of 11 immune-related genes, *TRB*, *CD4*, *CD8*, *FOXP3*, *PD-1*, *OX40*, *GZMA*, *PRF1*, *PD-L1*, *IL10*, and *TIM3* normalized by *GAPDH* expression levels in 8 SCCHN tumors. Correlation analysis of expression levels: *GZMA* to *FOXP3* **(B)** and *OX40* to *GZMA* **(C)**.

To understand how the high somatic mutational load caused by the MMR deficiency or the DNA polymerase mutation would affect anti-tumor immunity or pro-tumorigenic network in the tumor microenvironment, we examined the expression profiles of 11 immune-related genes in the 8 tumors, including *CD4*, *CD8*, *TCR*β (*TRB*), granzyme A (*GZMA*), perforin 1 (*PRF1*), *OX40*, *FOXP3*, *IL10*, *TIM3*, PD-1 ligand-1 (*PD-L1*), and *PD-1* as shown in a heatmap in Figure 1A. We found that all tumors we examined in this study showed a higher expression level of *CD8* than *CD4*. We also identified a high expression level of *FOXP3*, a marker of regulatory T cells, suggesting a significant proportion of CD4^+^ T cells in the tumor microenvironment may belong to T cells with immune suppressive functions. However, we observed no significant difference in expression levels of any of these 11 genes between high mutational and other tumors. However, a tendency that higher *GZMA/TRB* and *CD8/TRB* ratios exist in the tumors with higher mutational burden was observed (*P*=0.053, *P*=0.044, respectively, data are not shown). We also found the significant correlation between the expression levels of *GZMA* and *FOXP3* (*R*=0.68, *P*=0.001, Figure 1B), indicating the activation of immune suppressive system as a negative feedback manner to protect the tumor cells from anti-tumor immunity. There was also a correlation between *OX40* and *GZMA* (*R*=0.51, *P*=0.002, Figure 1C), suggesting co-activation of helper and cytotoxic T cells in the microenvironment of these tumors.

### Comparison of TCR repertoire between TILs in original tumor tissues and *in vitro* expanded TILs

To assess the TCR repertoire of TILs in the tumors and *in vitro*-expanded TILs of patients with SCCHN, we isolated RNAs from the tumors and expanded TILs of these 8 patients, and performed TCR-α and TCR-β sequencing by amplifying the TCR-α and -β chain cDNA using a MiSeq Illumina sequencing protocol. For TILs in the tumors, we obtained an average of 258,316 ± 38,985 total sequence reads (average ± one standard deviation (SD)) for TCR-α and 752,145 ± 203,930 (average ± one SD) for TCR-β per tumor, which consisted of the entire V, (D for beta chain), J and C segments. 11,155 ± 3,028 (average ± one SD) unique TCR-α complementarity determining region 3 (CDR3) clonotypes and 39,919 ± 13,123 (average ± one SD) unique TCR-β CDR3 clonotypes were identified.

For *in vitro* expansion of TILs, we successfully expanded TILs from 7 of 8 tumors and finally obtained T lymphocytes with a range of 5.1×10^7^ - 2×10^9^ cells. Flow cytometry analysis of expanded lymphocytes in 5 tumors (started from the non-sorted single cells) revealed that the proportions of CD4^+^ cells ranged from 25.7% to 84.0% and those of CD8^+^ cells ranged from 11.4% to 67.5%. For patient B2 and B4, we cultured CD8^+^ TILs sorted out from the single cell suspension. The characteristics of *in vitro* TIL culture including the composition of CD4^+^/CD8^+^ populations of expanded TILs and the numbers of potential HLA-restricted neoantigens are summarized in Table 2.

**Table 2:** TILs culture *in vitro* and CD4/CD8 composition of expanded TILs in 8 SCCHN patients

Patient No	No. of missense mutations	No. of HLA-A restricted neoantigen candidates (the affinity of <500 nM)	No. of cells at the starting point	No. of expanded TILs	Expanded TILs	
					CD4 (%)	CD8 (%)
B1	51	7	N/A	N/A	N/A	N/A
B2	1779	251	1×10^5^CD8^+^cells	8.0×10^7^	N/A	93.0
B3	11	0	6×10^6^ single cells	9.0×10^8^	25.7	67.5
B4	48	4	1×10^5^CD8^+^cells	8.3×10^7^	N/A	95.7
B5	4290	532	6×10^6^ single cells	2.4×10^8^	84.0	11.4
B6	49	19	6×10^6^ single cells	5.1×10^7^	34.5	42.3
B7	78	14	6×10^6^ single cells	2.0×10^9^	39.5	56.5
B8	901	273	6×10^6^ single cells	1.8×10^9^	34.4	58.0

N/A: Not applicable.

**Figure 2:**
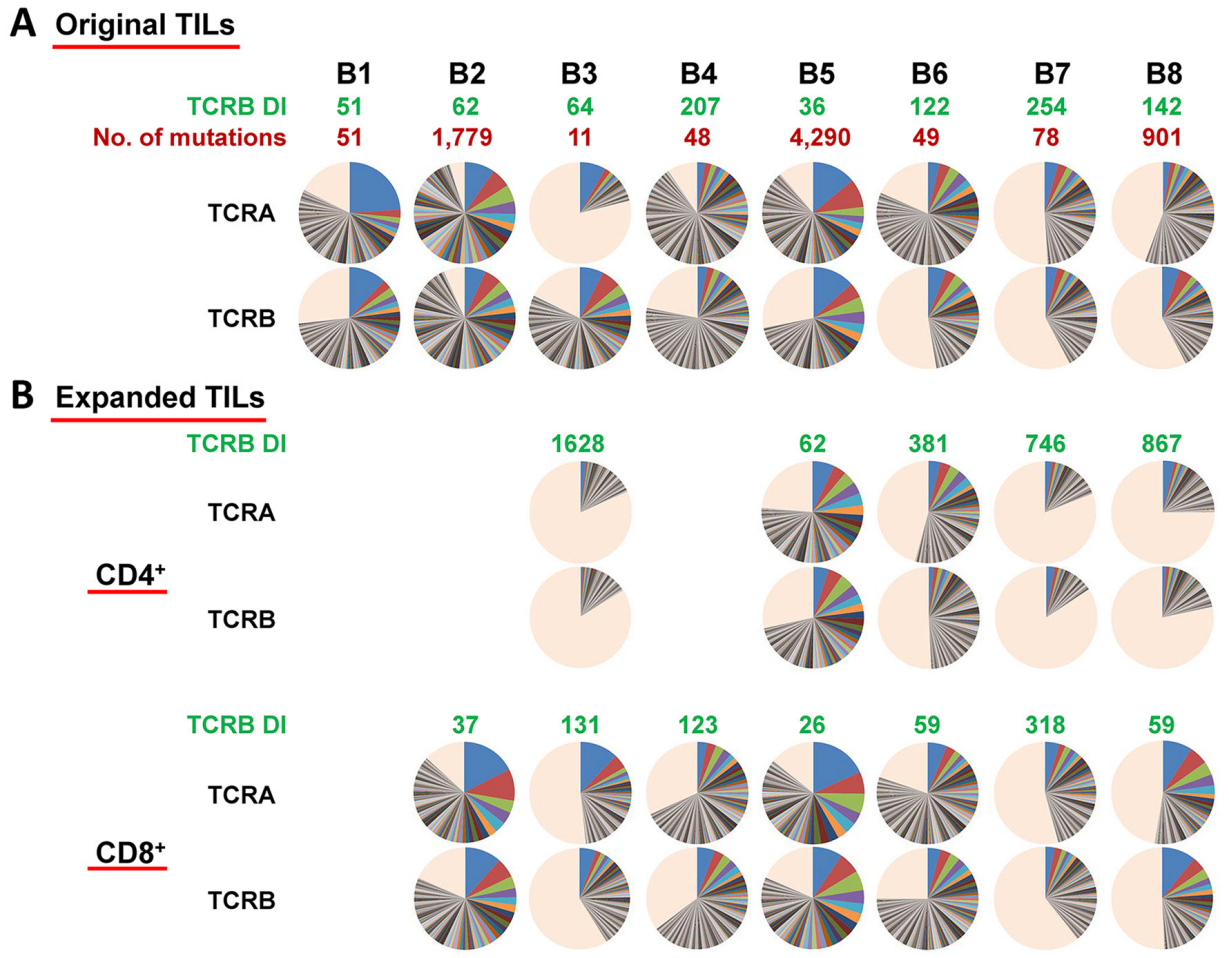
**(A)** Distribution of both TCR-α and TCR-β CDR3 unique clonotypes, the numbers of mutations in individual tumors as well as TCR-β DIs in original TILs in SCCHN tumors. **(B)** Distribution of both TCR-α and TCR-β CDR3 unique clonotypes and TCR-β DIs in expanded CD4^+^/CD8^+^ TILs. Pie charts depict clonotypes with 0.1% frequency or higher in each patient. Common colors among different pie charts do not represent identical clonotypes. The light orange portion of each pie chart contains clonotypes with less than 0.1% frequency.

For TCR sequences of *in vitro* expanded TILs, five CD4^+^ populations and seven CD8^+^ populations, we obtained an average of 462,233 ± 86,151 total sequence reads (average ± one SD) for TCR-α and 544,485 ± 80,265 (average ± one SD) for TCR-β per sample. We identified 25,449 ± 4,243 and 35,183 ± 4,876 (average ± one SD) unique CDR3 clonotypes of TCR-α and TCR-β, respectively. We then compared the clonality (shown by pie charts) and diversity index (DI) of TILs in the original tissues and expanded T cell populations as shown in Figure 2A and 2B. It was obvious that there was no correlation between the mutational load and their clonal expanded patterns. However, the TCR-β DIs of expanded CD4^+^ populations increased significantly (*P*=0.012, data not shown), suggesting less clonal expansion of CD4^+^ populations, whereas those of CD8^+^ populations remained similar with the corresponding original TILs.

**Figure 3:**
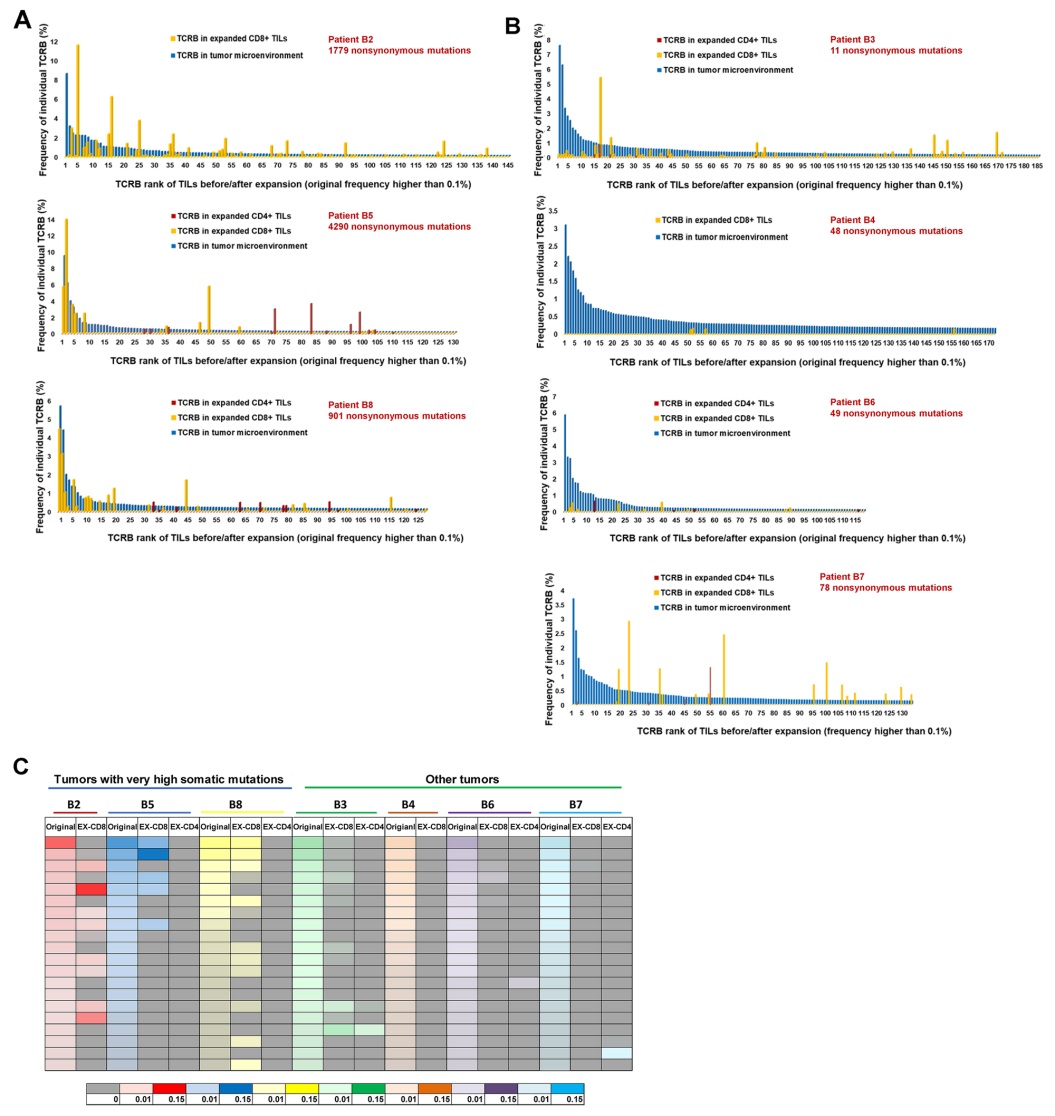
Comparison of TCR-β repertoire between TILs in original tumors and those expanded *in vitro* **(A)** Comparison of TCR-β clonotypes of TILs before and after expansion in 3 tumors with very high mutational load. **(B)** Comparison of TCR-β clonotypes of TILs before and after expansion in remaining 4 tumors. The blue bars represent the frequencies (ordered by the frequencies from highest to lowest) of the common CDR3 clonotypes (the frequency of 0.1% or higher in all mapped TCR reads) in TILs in the original tumors (a mixture of CD4^+^ and CD8^+^ cells). The orange bars represent the frequencies of CDR3 clonotypes in expanded CD8^+^ populations and the red bars represent those in the expanded CD4^+^ populations. **(C)** Heatmap showing changes between original top 20 clones before and after *in vitro* expansion. For each patient, TCR-β repertoire data of original TILs (represented by original), expanded CD8^+^ TILs (represented by EX-CD8), expanded CD4^+^ TILs (represented by EX-CD4) are listed. The frequencies of individual CDR3 clonotypes are indicated by graduation of colors and different colors on the bottom of the heatmap indicate individual patients.

We then focused on the individual clonotypes in these samples. Figure 3A and 3B showed the frequencies of the TCRB clonotypes, which were dominantly observed in TILs in the original tissues (the frequency of 0.1% or higher), in the expanded CD4^+^ and CD8^+^ populations; Figure 3A demonstrated the results of tumors with very high mutational load, B2, B5 and B8, and Figure 3B showed those for the remaining tumors. The blue bars represent the frequencies (from highest to lowest) of the dominant TCRB CDR3 clonotypes in TILs in the original tumors (a mixture of CD4^+^ and CD8^+^ cells). The orange bars represent the frequencies of the TCRB CDR3 clonotypes in the expanded CD8^+^ populations and the red bars represent those in the expanded CD4^+^ populations. All combinations of V, J and CDR3 sequences of TCRB clonotypes with frequency 0.1% or higher in each tumor are summarized in Supplementary Table 2. The comparison results of TCRA CDR3 clonotypes between original TILs and *in vitro* expanded ones, similar with those of TCRB CDR3 clonotypes, are shown in Supplementary Figure 1.

Interestingly, the TCR clonotypes of expanded CD8^+^ populations overlapped significantly to those of TILs in the original tumors in three cases carrying very high numbers of somatic mutations while the TCR repertoires of CD4^+^ populations in these three tumors as well as those of expanded TILs in the remaining four tumors drastically different from those of TILs in the original tumors. In these three cases, the majority of dominantly-observed TCR clonotypes in the original tissues were also well represented in the TCR reads in the expanded CD8^+^ populations. As summarized in a heatmap in Figure 3C, we present the comparison of the top 20 most-common TCR clonotypes in TILs in the original tissues with the respective expanded CD8^+^ and CD4^+^ populations. This data clearly indicates that most of the dominant clonotypes in the original tumors disappeared after *in vitro* expansion of T cells except for CD8^+^ populations in the three cases with the very high mutational burden. We then calculated the sum of the frequencies of top 20 TCRA/TCRB CDR3 clonotypes of TILs in both groups before and after expansion, and found that in the extremely high mutational load group, the sums of top 20 clones TCRA/TCRB remained to be around 15 to 40% of total CD8^+^ TILs after expansion *in vitro (P*=0.53 for TCRA and *P*=0.22 for TCRB by unpaired *t* test). On the other hand, in the low mutational load group, the sums of top 20 TCRA/TCRB clones decreased significantly from 20-50% to 0-8% of the total CD8^+^ TILs after expansion (*P*=0.0004 for TCRA and *P*=0.0015 for TCRB by unpaired *t* test) (Supplementary Figure 2).

## DISCUSSION

The scientific rationale of adoptive TIL immunotherapy stands on the consensus that a fraction of TILs in the tumor microenvironment is a key mediator of anti-tumor activity through recognition of the tumor-specific neoantigens (and possibly shared antigens) presented on tumor cells [14]. However, since clinical responses of TIL treatment are limited [15–17], we pursued an in-depth characterization to better understand the nature of TILs in the tumor microenvironment as well as expanded TILs for further improvement of adoptive TIL immunotherapy in patients with SCCHN.

In this study, we attempted to figure out the similarities/differences in TCR clonotypes of TILs in the original tumors and in *in vitro* expanded TILs in eight SCCHN tumors. In the three cases showing very high mutational load, we identified mutations in mismatch repair genes, *MSH2* or *MSH4*, in two cases and a missense mutation in a DNA polymerase gene, *POLE*, in one case to explain the underlying mechanism of these unusually high somatic mutations. Mutations in these genes are known to cause the dysfunctions in the DNA repair process or in the DNA replication process [18–20], resulting in an unusually high number of somatic mutations. However, since mutations in these genes have not been described as a common event in SCCHN, further investigation into MMR deficiency/DNA polymerase mutation in SCCHN may be warranted to better define the clinical and biologic characteristics as patients may potentially benefit from immunotherapy, including both checkpoint blockades as well as adoptive cellular therapies.

Interestingly, we found TCR clonotypes in TILs in the original tumors and in *in vitro* expanded TILs to differ markedly from each other, while in these three tumors with very high mutational burden, we observed significant overlap of clonotypes between TILs in the original tumors and expanded CD8^+^ populations. In the *in vitro* expansion process of TILs, there is no method to control proliferation of T cells with specific clonotypes, or to routinely monitor or predict which T cell subclones would preferentially expand. Only 27% of patients, who undergo tumor resection for TIL preparation, eventually received TIL therapy [21]. TCR repertoires, which represent the composition of various TIL subclones, may serve as good indicators to evaluate the changes of TILs during the culture process. The comparison of TCR repertoire or expression levels of immune-related genes in the original TILs and expanded TILs may provide useful information for defining promising T cells that are most conducive to T cell treatment.

This is the first report showing the composition of clonotypes of expanded TILs derived from tumors with the very high mutational burden is quite similar with that of TILs in the original tumor microenvironment. The data suggests that tumors with higher number of somatic mutations have a higher chance to generate a higher number of strongly immunogenic neoantigens and possibly result in the stronger clonal expansion of activated T cells with high cytolytic activity. In our present study, we also performed the prediction of possible neo-antigenic peptides using the WES and transcriptome data, and identified a very strong correlation between the mutational load and the numbers of neoantigen candidates (*R*=0.96), supporting our hypothesis that the higher number of somatic missense mutations could generate a higher number of neoantigens. However, since we didn’t examine the autologous anti-tumor activity of expanded TILs against cancer cells as reported previously [22, 23], we are unable to make a solid conclusion.

The reason for the big differences in TCR clonotypes between TILs in the original tumor tissues and expanded TILs is unclear. One possibility is that the single suspension cell populations from tumor tissues did not reflect accurately the entire cell population of tumors (cell preparation bias). However, we think that this possibility is less likely because we observed the common clones in the original TILs and expanded CD8^+^ cells in the three cases with very high mutational load. The other possibility is the genetic heterogeneity of tumors as we reported previously; we found the presence of intra-tumor heterogeneity of T cell repertoire due to intra-tumor mutational heterogeneity [24, 25]. Hence, we examined T cell repertoire of single cell suspensions of original tumors, which were used for *in vitro* expansion, in six cases. The results indicated that TCR clonotypes of single cell suspensions were similar with those of original tumor tissues (using RNAs isolated from bulk original tissues) (data are not shown), indicating little possibility of intra-tumor heterogeneity and supporting an idea that a subset of T cells in the original tissues could expand preferentially during *in vitro* culture.

In conclusion, although TIL therapy has been explored in clinical trials for many years, we need to accumulate the information of T cell repertoires of TILs in the original tumor tissues and TILs infused to patients to better predict and improve the clinical response of this type of immunotherapy.

## MATERIALS AND METHODS

### Materials

8 patients with SCCHN were previously treated with or without platinum-based chemotherapy at the University of Chicago. The clinicopathological characteristics of these patients are summarized in Table 1. Fresh surgically-resected tumors from 8 patients (B1-B8) were obtained from November 2016 till April 2017. Peripheral blood or normal adjacent tissue (pathology reviewed, e.g. uninvolved lymph nodes, muscle, etc.) were collected as normal controls. The study protocol was approved by the Institutional Review Board of the University of Chicago (approval number 8980, 13-0797, and 13-0526). All patients provided a written informed consent for research.

### Extraction of DNAs and RNAs

Genomic DNAs and total RNAs were extracted from tumor and adjacent normal tissues using the AllPrep DNA/RNA mini kit (Qiagen, Catalog number 80207), genomic DNAs were extracted from peripheral blood using QIAamp DNA Blood Midi Kit (Qiagen, Catalog number 51183). RNAs from expanded TILs were extracted using PicoPure RNA Isolation kit (Life Technologies, Catalog number KIT0204).

### Whole-exome sequencing and transcriptome analysis

Whole-exome libraries were built up using SureSelectXT Human All Exon V5 kit (Agilent Technologies, Catalog number 5190-6208) and sequenced by 100-bp paired-end reads on HiSeq2500 Sequencer (Illumina, San Diego, CA, USA). The obtained sequence data were analyzed using in house pipeline as described previously [26]. Briefly, the reads were mapped to the human reference genome GRCh37/hg19 using Burrows-Wheeler Aligner (BWA), then possible PCR duplicates were removed with Picard tool (http://broadinstitute.github.io/picard/). Read pairs with a mapping quality of < 30 and with mismatches more than 5% of read length were removed either. Somatic variants (single nucleotide variations (SNVs) and indels) were called using the following parameters, (i) base quality ≥ 15, (ii) sequence depth ≥ 10, (iii) variant depth ≥ 4, (iv) variant frequency in tumor ≥10%, (v) variant frequency in normal < 2%, and (vi) Fisher P value < 0.05 [27]. SNVs and indels were annotated based on RefGene using ANNOVAR [28].

### Isolation and expansion of TILs

TILs isolation and culture were performed following a modified traditional TILs culture protocol [22]. Briefly, fresh tumors were chopped until they are small chunks; less than 1×1 mm, digested with collagenase mixture in 37°C shaking incubator for 1-4 h. The single cell suspension was collected and cultured for two weeks with 1000 IU/mL IL2 (R&D Systems, Catalog number 202-IL-050) stimulation in RetroNectin-coated flask (Takara Bio, Catalog number T100A). CD4^+^ and CD8^+^ subgroups of expanded TILs were isolated with Dynabeads CD4/CD8 positive isolation kit (ThermoFisher Scientific, Catalog numbers 11331D and 11333D).

### TCR sequencing

The libraries for TCR sequencing were prepared following the protocol as described previously [29]. Briefly, quality of total RNAs extracted from tumors and expanded TILs were evaluated with TapeStation 2200 (Agilent Technologies). 5’ rapid amplification of cDNA end adapter was added during the cDNA synthesis using SMART cDNA library construction kit (Clontech). TCR-α and TCR-β sequences were amplified using a forward primer for the SMART adapter and a reverse primer specific to the TCR constant region. Then Illumina sequence adapter with barcode sequences were added using the Nextera XT Index kit (Illumina, Catalog numbers FC-131-2001, FC-131-2002, FC-131-2003).

The final prepared libraries were sequenced by 300-bp paired-end reads on the Illumina MiSeq platform using missed Reagent v3 600-cycles kit (Illumina, Catalog number MS-102-3001). Sequencing data analysis was performed using Tcrip as described previously [30]. Briefly, sequencing reads were mapped to the TCR reference sequences obtained from IMGT/GENE-DB (http://www.imgt.org) using Bowtie2 aligner (version 2.1.0), and after decomposition of sequencing reads into V, (D) and J segments, CDR3 were searched. The inverse Simpson’s diversity index (DI) were used to evaluate the TCR-β clonality [31].

### Gene expression analysis

mRNA expression levels of 11 immune-related genes, *TRB*, *CD4*, *CD8*, *FOXP3*, *PD-1*, *OX40*, *GZMA*, *PRF1*, *PD-L1*, *IL10*, and *TIM3* were measured by quantitative real-time RT-PCR using TaqMan gene expression assays (ThermoFisher Scientific, Catalog number 4331182). All mRNA expression levels were normalized to *GAPDH* expression level (Hs02758991_g1).

### Statistical analysis

Pearson correlation (R) was used to analyze the association between all examined parameters. Unpaired t test analysis was performed using Prism 7 (GraphPad software, La Jolla, CA), *P* < 0.05 was considered statistically significant.

## SUPPLEMENTARY MATERIALS FIGURES AND TABLES





## Abbreviations

CDR3: complementarity determining region 3
DI: diversity index
MMR: mismatch repair
PD-1: programmed death-1
PD-L1: PD-1 ligand-1
SCCHN: squamous cell carcinoma of head and neck cancer
TCR: T-cell receptor
TILs: tumor-infiltrating lymphocytes
