# Supplementary material for: Similarity and difference in tumor-infiltrating lymphocytes in original tumor tissues and those of in vitro expanded populations in head and neck cancer
Source: Oncotarget. 2017 Dec 19;9(3):3805–14. doi: 10.18632/oncotarget.23454 (PMC5790501; doi:10.18632/oncotarget.23454)
Supplement: Supplementary file 1 [file oncotarget-09-3805-s001.pdf]

Similarity and difference in tumor-infiltrating lymphocytes in original tumor tissues and those of *in vitro* expanded populations in head and neck cancer

SUPPLEMENTARY MATERIALS

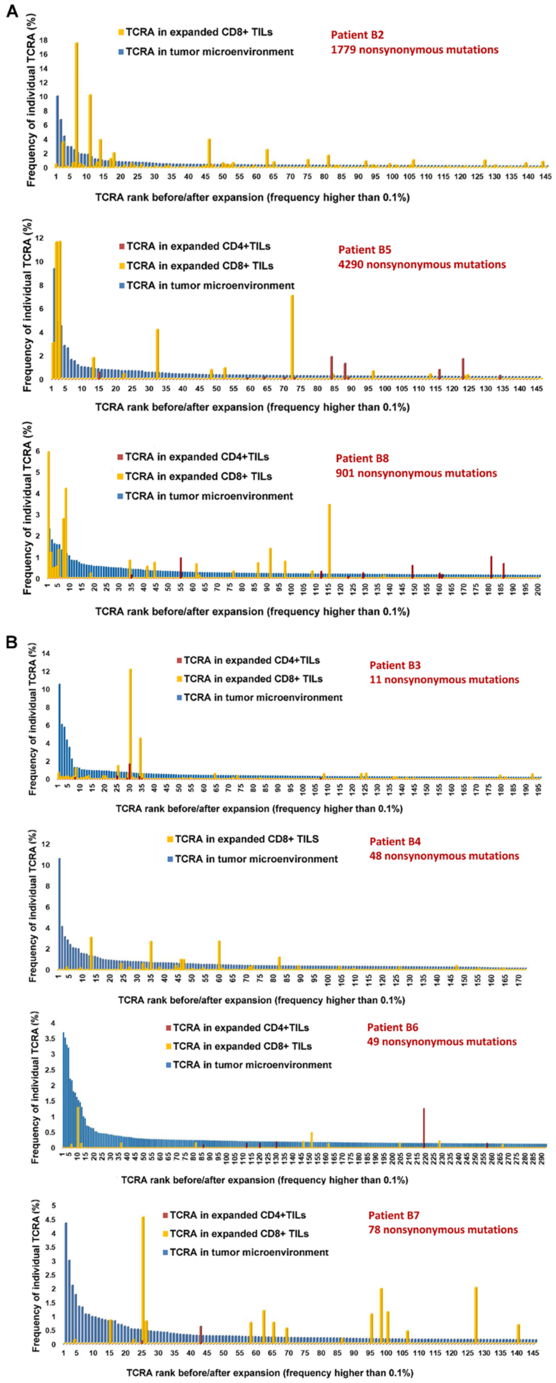

Supplementary Figure 1: Comparison of TCR-α repertoire between TILs in original tumors and those expanded *in vitro*. **(A)** Comparison of TCR-α clonotypes of TILs before and after expansion in 3 tumors with very high mutational load. **(B)** Comparison of TCR-α clonotypes of TILs before and after expansion in 4 remaining tumors. The blue bars represent the frequencies (ordered by the frequencies from highest to lowest) of the common CDR3 clonotypes (the frequency of 0.1% or higher in all mapped TCR reads) in TILs (a mixture of $CD4^+$ and $CD8^+$ cells) in the original tumors. The orange bars represent the frequencies of the CDR3 clonotypes in the expanded

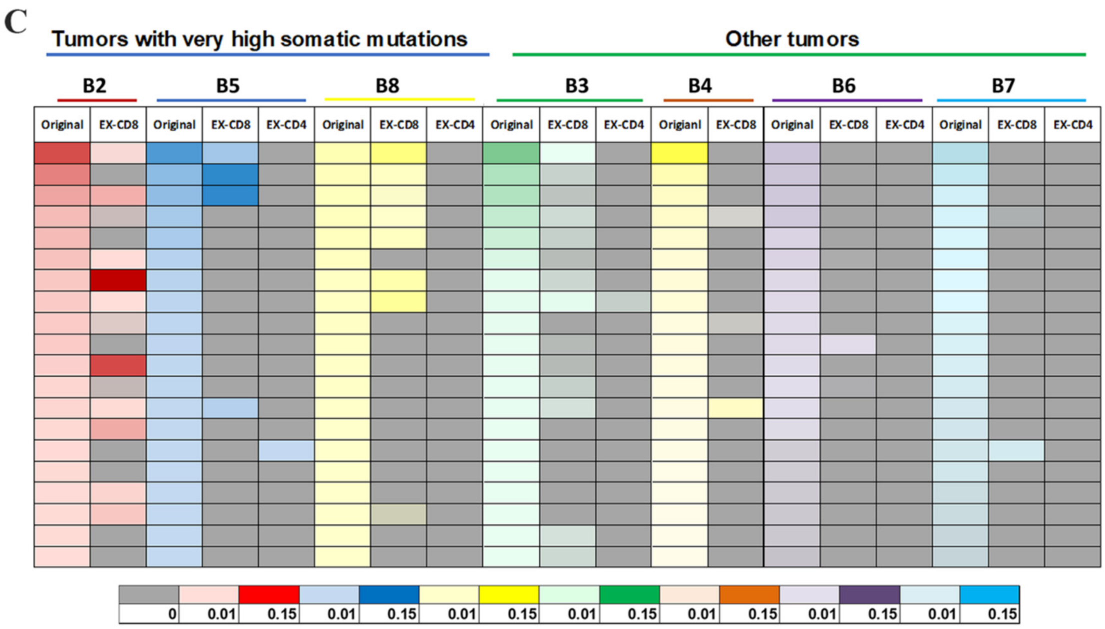

Supplementary Figure 1: (Continued) CD8+ populations and the red bars represent those in the expanded CD4+ populations. **(C)** Alteration heatmap of original top 20 clones before and after *in vitro* expansion. For each patient, TCR-α repertoire data of original TILs (represented by original), expanded CD8+ TILs (represented by EX-CD8), expanded CD4+ TILs (represented by EX-CD4) are listed. The frequencies of individual CDR3 clonotypes are indicated by graduation of colors and different colors on the bottom of the heatmap indicate individual patients.

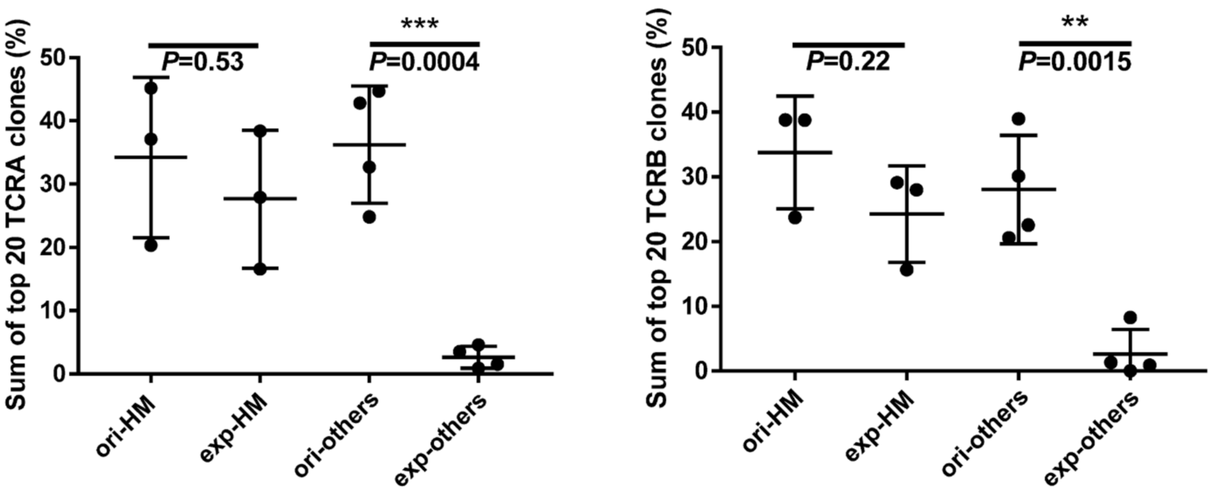

Supplementary Figure 2: Alteration of the sum of top 20 TCRA/TCRB CDR3 clonotypes before and after *in vitro* expansion in 3 tumors with very high mutational load and the remaining 4 tumors. The sums of top 20 TCRA/TCRB clonotypes of TILs in high mutation tumors (HM) and the remaining 4 tumors (others) are presented separately; "ori-HM" and "ori-others" represent TILs in original tumors. "exp-HM" and "exp-others" represent TILs after expansion. *P*-values were calculated for the difference between the sums of pre- and post-expansion groups by an unpaired *t* test.

Supplementary Table 1: The mutated genes related with DNA damage repair and DNA replication process in patients B2, B5, B8

Patients	Gene	Chromosomal location	Mutations	Amino acid substitution	Whole-exome data			RNA sequencing data		
					Reference reads	Variant reads	Variant frequency	Reference reads	Variant reads	Variant frequency
**B2**	*MSH2*	47643504	G to A	G338R	77	14	15.00%	14	27	65.90%
	POLH	43581878	C to T	P576S	121	18	13.00%	7	6	46.20%
	PER1	8048126	C to T	G802S	60	12	17.00%	3	10	76.90%
	TP53	7577608	C to G	V93L	39	12	23.50%	8	4	33.30%
	ATR	142212098	G to A	P1985L	83	14	14.40%	9	1	10.00%
	SHPRH	146275987	C to T	E158K	86	16	15.70%	0	0	0.00%
**B5**	*POLE*	133249800	G to A	H475Y	20	26	56.50%	3	5	62.50%
	SMUG1	54577462	G to A	P88L	64	15	19.00%	55	22	30.00%
	PNKP	50368448	G to A	P145L	35	51	59.30%	35	44	55.70%
	PER1	8046679	G to A	P993S	15	6	28.50%	70	13	15.70%
	MLH3	75515476	G to A	R295W	91	28	23.50%	2	0	0.00%
	FANCM	45645402	C to T	L1149F	125	25	16.70%	3	0	0.00%
	RNF168	196202112	G to A	S251F	78	21	21.20%	23	0	0.00%
**B8**	*MSH4*	76262710	C to T	P14S	82	17	17.20%	0	0	0.00%
	LIG3	33310250	G to A	G76R	90	16	15.10%	16	11	40.70%

Supplementary Table 2: Combinations of V, J and CDR3 sequences of TCR-β clonotypes with frequency 0.1% or higher in each tumor.

See Supplementary File 1
