# Supplementary material for: Similarity and difference in tumor-infiltrating lymphocytes in original tumor tissues and those of in vitro expanded populations in head and neck cancer
Source: Oncotarget. 2017 Dec 19;9(3):3805–14. doi: 10.18632/oncotarget.23454 (PMC5790501; doi:10.18632/oncotarget.23454)
Supplement: Supplementary file 2 [file oncotarget-09-3805-s002.docx]

**Supplementary Table 2:** Combinations of V, J and CDR3 sequences of TCR-β clonotypes with frequency 0.1% or higher in each tumor

| **TCRB repertoire in tumor B2 (0.1% or higher)** | | | | | | | |
| --- | --- | --- | --- | --- | --- | --- | --- |
| **No.** | **Vseg** | **Jseg** | **CDR3** | **No.** | **Vseg** | **Jseg** | **CDR3** |
| **1** | TRBV28*01 | TRBJ2-7*01 | CASSIGQTTYEQYF | **74** | TRBV27*01 | TRBJ2-7*01 | CASYSSSYEQYF |
| **2** | TRBV4-3*01 | TRBJ2-7*01 | CASSQGSGLAGGEQYF | **75** | TRBV27*01 | TRBJ2-7*01 | CASSLGTPFMQYF |
| **3** | TRBV5-1*01 | TRBJ1-1*01 | CASRVEGAWTEAFF | **76** | TRBV7-2*01 | TRBJ2-1*01 | CASSLTSGPYNEQFF |
| **4** | TRBV6-5*01 | TRBJ2-7*01 | CASSYSTSGSSYEQYF | **77** | TRBV27*01 | TRBJ2-7*01 | CASSLVQAYEQYF |
| **5** | TRBV14*01 | TRBJ2-7*01 | CASRPRGLYEQYF | **78** | TRBV14*01 | TRBJ2-1*01 | CASSQAPGADNEQFF |
| **6** | TRBV20-1*01 | TRBJ1-2*01 | CSARSGANYGYTF | **79** | TRBV10-3*01 | TRBJ2-3*01 | CATTTSGRATDTQYF |
| **7** | TRBV5-1*01 | TRBJ2-1*01 | CASSLAEQGFDEQFF | **80** | TRBV4-1*01 | TRBJ2-7*01 | CASSQGIIDVGEQYF |
| **8** | TRBV7-2*01 | TRBJ2-7*01 | CASSPDGTTGYEQYF | **81** | TRBV28*01 | TRBJ2-3*01 | CASSFLAGTDTQYF |
| **9** | TRBV7-9*01 | TRBJ2-1*01 | CASSLTQGVWNEQFF | **82** | TRBV27*01 | TRBJ2-1*01 | CASSLYSYNEQFF |
| **10** | TRBV3-1*01 | TRBJ2-2*01 | CASSQDPGRAGELFF | **83** | TRBV6-5*01 | TRBJ2-3*01 | CASSYETSGTRDTQYF |
| **11** | TRBV10-2*01 | TRBJ2-2*01 | CASRVENTGELFF | **84** | TRBV28*01 | TRBJ2-3*01 | CASSFVSGTDTQYF |
| **12** | TRBV7-6*01 | TRBJ2-7*01 | CASSLAADFSYEQYF | **85** | TRBV20-1*01 | TRBJ2-2*01 | CSGGGYTGELFF |
| **13** | TRBV7-9*01 | TRBJ2-6*01 | CASIGVSGANVLTF | **86** | TRBV27*01 | TRBJ2-2*01 | CASLPGLQGAETGELFF |
| **14** | TRBV27*01 | TRBJ2-2*01 | CASRLAGQGAGELFF | **87** | TRBV29-1*01 | TRBJ2-5*01 | CSVEQGAGETQYF |
| **15** | TRBV11-3*01 | TRBJ2-1*01 | CASSLAGPSSYNEQFF | **88** | TRBV6-6*01 | TRBJ1-1*01 | CASSPLGAGDAEAFF |
| **16** | TRBV5-1*01 | TRBJ2-1*01 | CASSLAAGGNEQFF | **89** | TRBV7-9*01 | TRBJ2-2*01 | CASSSWTGNTGELFF |
| **17** | TRBV20-1*01 | TRBJ1-1*01 | CSALEGTSGAFF | **90** | TRBV7-8*01 | TRBJ2-4*01 | CASSLGQENIQYF |
| **18** | TRBV29-1*01 | TRBJ1-2*01 | CSVEQGNNHGYTF | **91** | TRBV7-8*01 | TRBJ2-1*01 | CASSPLVNEQFF |
| **19** | TRBV19*01 | TRBJ2-1*01 | CASSIGGVEQFF | **92** | TRBV20-1*01 | TRBJ1-5*01 | CSARDNTGNQPQHF |
| **20** | TRBV12-5*01 | TRBJ1-1*01 | CASGYSGLEAFF | **93** | TRBV3-1*01 | TRBJ2-3*01 | CASSQVSEGGTDTQYF |
| **21** | TRBV20-1*01 | TRBJ2-4*01 | CSARSRAALASNIQYF | **94** | TRBV6-5*01 | TRBJ2-1*01 | CASSYSEQQFF |
| **22** | TRBV28*01 | TRBJ2-5*01 | CASSSPRDPGGSETQYF | **95** | TRBV5-1*01 | TRBJ1-1*01 | CASSLDLSGLEAFF |
| **23** | TRBV7-7*01 | TRBJ2-1*01 | CASSLTAGAEQFF | **96** | TRBV9*01 | TRBJ1-5*01 | CASSVDLGPQPQHF |
| **24** | TRBV27*01 | TRBJ2-1*01 | CASGGDGTYNEQFF | **97** | TRBV2*01 | TRBJ1-5*01 | CASGREALGNQPQHF |
| **25** | TRBV7-9*01 | TRBJ1-1*01 | CASSFPLGGSMNTEAFF | **98** | TRBV19*01 | TRBJ2-1*01 | CASSTETYNEQFF |
| **26** | TRBV29-1*01 | TRBJ2-2*01 | CSGYRNTGELFF | **99** | TRBV15*01 | TRBJ2-7*01 | CATSWSGSSYEQYF |
| **27** | TRBV4-3*01 | TRBJ2-7*01 | CASSQDPPRGGTYEQYF | **100** | TRBV6-6*01 | TRBJ2-4*01 | CASSYNHLAAGRAKNIQYF |
| **28** | TRBV7-9*01 | TRBJ2-1*01 | CASRPTGQGTYNEQFF | **101** | TRBV30*01 | TRBJ2-1*01 | CAWSYGSSSYNEQFF |
| **29** | TRBV11-2*01 | TRBJ2-5*01 | CASRTSGRQRETQYF | **102** | TRBV9*01 | TRBJ1-2*01 | CASSVDFNGYTF |
| **30** | TRBV27*01 | TRBJ2-7*01 | CASSLKQSYEQYF | **103** | TRBV7-3*01 | TRBJ2-7*01 | CASSSALGASAYEQYF |
| **31** | TRBV7-9*01 | TRBJ2-1*01 | CASSAQGSPGRRYNEQFF | **104** | TRBV27*01 | TRBJ2-1*01 | CASSLSRQNEQFF |
| **32** | TRBV20-1*01 | TRBJ1-6*01 | CSAPRAGQTLSAYNSPLHF | **105** | TRBV27*01 | TRBJ2-7*01 | CASSPDSGGRSYEQYF |
| **33** | TRBV20-1*01 | TRBJ2-7*01 | CSARDPVLSSGFTYEQYF | **106** | TRBV5-1*01 | TRBJ2-1*01 | CASSLYGNEQFF |
| **34** | TRBV10-2*01 | TRBJ1-1*01 | CASSVDGMNTEAFF | **107** | TRBV20-1*01 | TRBJ2-1*01 | CSARALGGHEQFF |
| **35** | TRBV2*01 | TRBJ2-7*01 | CASSEVAGVSYEQYF | **108** | TRBV5-1*01 | TRBJ2-2*01 | CASSSGLAGVGGELFF |
| **36** | TRBV5-5*01 | TRBJ1-2*01 | CASSLEAGDNYGYTF | **109** | TRBV19*01 | TRBJ1-2*01 | CASSIADTAGYTF |
| **37** | TRBV24-1*01 | TRBJ2-7*01 | CATSSRGGAVGYEQYF | **110** | TRBV29-1*01 | TRBJ1-5*01 | CSVEETGGNQPQHF |
| **38** | TRBV7-3*01 | TRBJ2-2*01 | CASSSPGLADPNTGELFF | **111** | TRBV20-1*01 | TRBJ2-1*01 | CSARVYGTSGRRSYNEQFF |
| **39** | TRBV4-1*01 | TRBJ2-1*01 | CASSLNHGYNEQFF | **112** | TRBV27*01 | TRBJ1-5*01 | CASSLLGGGSNQPQHF |
| **40** | TRBV29-1*01 | TRBJ2-1*01 | CSVPLASLNEQFF | **113** | TRBV5-5*01 | TRBJ2-7*01 | CASSFGGLDEQYF |
| **41** | TRBV7-6*01 | TRBJ2-2*01 | CASSLNLDSGELFF | **114** | TRBV10-2*01 | TRBJ1-4*01 | CASGEPYRGAGEKLFF |
| **42** | TRBV6-2*01/TRBV6-3*01 | TRBJ1-1*01 | CASRFRGSTEAFF | **115** | TRBV29-1*01 | TRBJ2-7*01 | CSVVQGGRQYF |
| **43** | TRBV7-8*01 | TRBJ2-5*01 | CASSLGDQGGTSGSQETQYF | **116** | TRBV7-8*01 | TRBJ2-4*01 | CASSTGADIQYF |
| **44** | TRBV2*01 | TRBJ1-2*01 | CASTPGQGDYGYTF | **117** | TRBV20-1*01 | TRBJ1-5*01 | CSATPNQPQHF |
| **45** | TRBV9*01 | TRBJ2-2*01 | CASSVYGSGGTGELFF | **118** | TRBV29-1*01 | TRBJ2-1*01 | CSVEDPIPSSYNEQFF |
| **46** | TRBV6-5*01 | TRBJ1-2*01 | CASSTTGDYGYTF | **119** | TRBV2*01 | TRBJ2-1*01 | CASSGPEQFF |
| **47** | TRBV10-2*01 | TRBJ1-6*01 | CASSDYETSPLHF | **120** | TRBV15*01 | TRBJ2-5*01 | CATSNFQETQYF |
| **48** | TRBV14*01 | TRBJ2-3*01 | CASSQSPGGTQYF | **121** | TRBV30*01 | TRBJ2-2*01 | CAWAIYGIGAGELFF |
| **49** | TRBV7-8*01 | TRBJ2-1*01 | CASSVGTGGYNEQFF | **122** | TRBV24-1*01 | TRBJ1-2*01 | CATSDFAGLEANYGYTF |
| **50** | TRBV7-9*01 | TRBJ1-3*01 | CASSYGQGTRSTIYF | **123** | TRBV27*01 | TRBJ2-7*01 | CASRTPAGLANSYEQYF |
| **51** | TRBV9*01 | TRBJ2-3*01 | CASSGHQGARTDTQYF | **124** | TRBV20-1*01 | TRBJ2-5*01 | CSAINWARLAGKTQYF |
| **52** | TRBV4-3*01 | TRBJ2-1*01 | CASSQDGNRYNEQFF | **125** | TRBV29-1*01 | TRBJ2-7*01 | CSVEYRGLSSYEQYF |
| **53** | TRBV6-5*01 | TRBJ2-7*01 | CASSSPRQEGLSYEQYF | **126** | TRBV6-2*01/TRBV6-3*01 | TRBJ2-2*01 | CASSLTLAVRTGELFF |
| **54** | TRBV27*01 | TRBJ2-7*01 | CASSLGAVSYEQYF | **127** | TRBV28*01 | TRBJ1-6*01 | CASSPPRGPLHF |
| **55** | TRBV2*01 | TRBJ2-7*01 | CASSEVSGSPYEQYF | **128** | TRBV24-1*01 | TRBJ2-1*01 | CATSDFGGTSGSYNEQFF |
| **56** | TRBV2*01 | TRBJ2-1*01 | CASRVLGSSYNEQFF | **129** | TRBV6-2*01/TRBV6-3*01 | TRBJ1-1*01 | CASSKVGDMNTEAFF |
| **57** | TRBV21-1*01 | TRBJ1-5*01 | CASSRKGRGQGQPQHF | **130** | TRBV5-6*01 | TRBJ2-5*01 | CASSYSTVYQETQYF |
| **58** | TRBV20-1*01 | TRBJ2-1*01 | CSARDISGGYEQFF | **131** | TRBV6-2*01/TRBV6-3*01 | TRBJ1-1*01 | CASSKVGDMNTEAFF |
| **59** | TRBV28*01 | TRBJ1-1*01 | CASSLAGTGHNGVF | **132** | TRBV5-6*01 | TRBJ2-5*01 | CASSYSTVYQETQYF |
| **60** | TRBV3-1*01 | TRBJ2-1*01 | CASSPYAPGSSYNEQFF | **133** | TRBV29-1*01 | TRBJ2-7*01 | CSVAYPNAPHEQYF |
| **61** | TRBV10-3*01 | TRBJ2-3*01 | CAISESGRSTDTQYF | **134** | TRBV27*01 | TRBJ2-7*01 | CASASGTSGAYEQYF |
| **62** | TRBV3-1*01 | TRBJ2-1*01 | CASSQDHNEQFF | **135** | TRBV27*01 | TRBJ1-2*01 | CASSDRGDGYHGYTF |
| **63** | TRBV11-2*01 | TRBJ1-4*01 | CASSLESLGWREKLFF | **136** | TRBV5-1*01 | TRBJ1-2*01 | CASSLVGTYGYTF |
| **64** | TRBV28*01 | TRBJ2-2*01 | CASSLNGELFF | **137** | TRBV7-3*01 | TRBJ2-1*01 | CASSHGVPNEQFF |
| **65** | TRBV19*01 | TRBJ2-1*01 | CASSIQAANNEQFF | **138** | TRBV4-2*01 | TRBJ2-5*01 | CASSQGTTSAETQYF |
| **66** | TRBV28*01 | TRBJ2-3*01 | CASSFGGSSYSTDTQYF | **139** | TRBV5-1*01 | TRBJ2-2*01 | CASSWDPLQNTGELFF |
| **67** | TRBV20-1*01 | TRBJ2-1*01 | CSLGGLAGKFNEQFF | **140** | TRBV10-3*01 | TRBJ2-7*01 | CAISDSSEREGHEQYF |
| **68** | TRBV19*01 | TRBJ2-6*01 | CASSRDWDISSGANVLTF | **141** | TRBV28*01 | TRBJ2-5*01 | CASSFRGAEKETQYF |
| **69** | TRBV7-2*01 | TRBJ1-1*01 | CASSLTGRTEAFF | **142** | TRBV14*01 | TRBJ2-5*01 | CASSQSGEMETQYF |
| **70** | TRBV5-1*01 | TRBJ2-7*01 | CASSLLWTGDEQYF | **143** | TRBV27*01 | TRBJ2-3*01 | CASSPVDHTDTQYF |
| **71** | TRBV20-1*01 | TRBJ1-5*01 | CSARDVLTAYNQPQHF | **144** | TRBV12-2*01 | TRBJ1-1*01 | CASRLDKRHGAEAFF |
| **72** | TRBV6-6*01 | TRBJ2-1*01 | CASSYSRGKYNEQFF | **145** | TRBV28*01 | TRBJ1-5*01 | CASSLFGTGNQPQHF |
| **73** | TRBV6-5*01 | TRBJ2-2*01 | CASSRTLAPNTGELFF |  |  |  |  |

| **TCRB repertoire in tumor B3 (0.1% or higher)** | | | | | | | | | |
| --- | --- | --- | --- | --- | --- | --- | --- | --- | --- |
| **No.** | **Vseg** | **Jseg** | **CDR3** | **No.** | | **Vseg** | **Jseg** | **CDR3** | |
| **1** | TRBV2*01 | TRBJ2-5*01 | CASSEAGAQETQYF | **94** | | TRBV12-4*01 | TRBJ2-7*01 | CASSLAGFGEQYF | |
| **2** | TRBV28*01 | TRBJ1-2*01 | CASSLRQGGVGYGYTF | **95** | | TRBV4-2*01 | TRBJ2-3*01 | CASSLGQPSTDTQYF | |
| **3** | TRBV7-3*01 | TRBJ2-1*01 | CASSLEAPGVEQFF | **96** | | TRBV28*01 | TRBJ1-2*01 | CASSLPTGADGYTF | |
| **4** | TRBV14*01 | TRBJ2-1*01 | CASSQRQEQFF | **97** | | TRBV5-6*01 | TRBJ2-3*01 | CASSFRADTQYF | |
| **5** | TRBV28*01 | TRBJ2-3*01 | CASRGTATDTQYF | **98** | | TRBV10-2*01 | TRBJ2-7*01 | CASSGAGGARTEQYF | |
| **6** | TRBV9*01 | TRBJ2-7*01 | CASSVGLAGAYEQYF | **99** | | TRBV28*01 | TRBJ2-1*01 | CASSFPTGGAWAYNEQFF | |
| **7** | TRBV2*01 | TRBJ2-7*01 | CASSGTGGEYEQYF | **100** | | TRBV30*01 | TRBJ2-1*01 | CAWSGGPVHEQFF | |
| **8** | TRBV27*01 | TRBJ2-7*01 | CASSFGQGGEQYF | **101** | | TRBV6-5*01 | TRBJ2-1*01 | CASSLSGGPNEQFF | |
| **9** | TRBV5-1*01 | TRBJ1-2*01 | CASSLEEGEDGYTF | **102** | | TRBV4-1*01 | TRBJ2-1*01 | CASSHPGLAVLEQFF | |
| **10** | TRBV7-2*01 | TRBJ1-1*01 | CASSLAVDGAKNTEAFF | **103** | | TRBV5-1*01 | TRBJ2-1*01 | CASSSLSGVFRTNNEQFF | |
| **11** | TRBV2*01 | TRBJ1-5*01 | CASSTTPTRGASNQPQHF | **104** | | TRBV28*01 | TRBJ1-1*01 | CASSFRTLNTEAFF | |
| **12** | TRBV9*01 | TRBJ1-2*01 | CASSPGDSPYGYTF | **105** | | TRBV13*01 | TRBJ1-2*01 | CASSVAYGYTF | |
| **13** | TRBV7-2*01 | TRBJ2-7*01 | CASSFGTSGGSYEQYF | **106** | | TRBV18*01 | TRBJ2-6*01 | CASSPTDREVSGANVLTF | |
| **14** | TRBV7-9*01 | TRBJ2-5*01 | CASSLLVAEETQYF | **107** | | TRBV18*01 | TRBJ2-1*01 | CASSQAGGDPYNEQFF | |
| **15** | TRBV7-9*01 | TRBJ2-1*01 | CASSPGGTYEQFF | **108** | | TRBV28*01 | TRBJ1-1*01 | CASSVRDGRLEAFF | |
| **16** | TRBV13*01 | TRBJ2-7*01 | CASSSRGEQYF | **109** | | TRBV10-3*01 | TRBJ2-1*01 | CAISESSTGWYNEQFF | |
| **17** | TRBV11-2*01 | TRBJ2-1*01 | CASRAESSRSYNEQFF | **110** | | TRBV6-2*01/TRBV6-3*01 | TRBJ2-1*01 | CASSYVNEQFF | |
| **18** | TRBV13*01 | TRBJ2-1*01 | CASSARNEQFF | **111** | | TRBV6-5*01 | TRBJ1-2*01 | CASRRLEANYGYTF | |
| **19** | TRBV30*01 | TRBJ2-7*01 | CAWRDWRGEQYF | **112** | | TRBV24-1*01 | TRBJ1-5*01 | CATSRGGGQPQHF | |
| **20** | TRBV9*01 | TRBJ2-2*01 | CASSAWTGPSNTGELFF | **113** | | TRBV2*01 | TRBJ2-5*01 | CASSEAPGRQETQYF | |
| **21** | TRBV7-8*01 | TRBJ2-7*01 | CASSYGQAYEQYF | **114** | | TRBV19*01 | TRBJ2-1*01 | CASSPLTAGKGNEQFF | |
| **22** | TRBV7-9*01 | TRBJ1-1*01 | CASSFGANTEAFF | **115** | | TRBV4-2*01 | TRBJ2-7*01 | CASSLRGGASYEQYF | |
| **23** | TRBV13*01 | TRBJ2-1*01 | CASSHNRAANEQFF | **116** | | TRBV7-9*01 | TRBJ2-7*01 | CASSAGDRGLEQYF | |
| **24** | TRBV13*01 | TRBJ2-3*01 | CASSSRDTQYF | **117** | | TRBV5-1*01 | TRBJ2-3*01 | CASSLEASGGVDTQYF | |
| **25** | TRBV27*01 | TRBJ2-6*01 | CASSLSPDGANVLTF | **118** | | TRBV3-1*01 | TRBJ1-2*01 | CASSQDRPHPTF | |
| **26** | TRBV27*01 | TRBJ2-7*01 | CASSRGQGKEQYF | **119** | | TRBV20-1*01 | TRBJ2-3*01 | CSARERGLATDTQYF | |
| **27** | TRBV5-1*01 | TRBJ2-3*01 | CASNTLVRSTDTQYF | **120** | | TRBV4-1*01 | TRBJ1-1*01 | CASSHPSTGETEAFF | |
| **28** | TRBV18*01 | TRBJ2-7*01 | CASSPSYEQYF | **121** | | TRBV7-9*01 | TRBJ2-1*01 | CASSLAGSGRALHINEQFF | |
| **29** | TRBV28*01 | TRBJ2-3*01 | CASSGTSGSTRGEDTQYF | **122** | | TRBV27*01 | TRBJ2-1*01 | CASSPGQGHEQFF | |
| **30** | TRBV7-9*01 | TRBJ2-1*01 | CASSFRERYEQFF | **123** | | TRBV27*01 | TRBJ1-2*01 | CASSLNRGYYGYTF | |
| **31** | TRBV5-5*01 | TRBJ2-7*01 | CASSGDWRGNYEQYF | **124** | | TRBV29-1*01 | TRBJ2-7*01 | CSGSAGTGDYEQYF | |
| **32** | TRBV5-1*01 | TRBJ2-3*01 | CASSPLARSTDTQYF | **125** | | TRBV25-1*01 | TRBJ1-4*01 | CASSADLLNEKLFF | |
| **33** | TRBV9*01 | TRBJ2-1*01 | CASFPGLAEVNEQFF | **126** | | TRBV15*01 | TRBJ2-7*01 | CATQRGAGEQYF | |
| **34** | TRBV28*01 | TRBJ2-6*01 | CASSLWGANVLTF | **127** | | TRBV3-1*01 | TRBJ2-7*01 | CASSQEGLYEQYF | |
| **35** | TRBV28*01 | TRBJ2-7*01 | CASSLRGQVEQYF | **128** | | TRBV2*01 | TRBJ2-5*01 | CASSEPGAIETQYF | |
| **36** | TRBV5-1*01 | TRBJ2-2*01 | CASSLATGQGISGELFF | **129** | | TRBV7-3*01 | TRBJ2-7*01 | CASSSRTSGKQSRDYEQYF | |
| **37** | TRBV7-9*01 | TRBJ1-1*01 | CASSLTGRTEAFF | **130** | | TRBV5-1*01 | TRBJ2-7*01 | CASSLASGDYEQYF | |
| **38** | TRBV7-3*01 | TRBJ2-2*01 | CASSLRTSGSWTGELFF | **131** | | TRBV24-1*01 | TRBJ2-1*01 | CATSDFASGSLGQFF | |
| **39** | TRBV28*01 | TRBJ2-2*01 | CASSGLNTGELFF | **132** | | TRBV12-4*01 | TRBJ1-1*01 | CASRATGGLNTEAFF | |
| **40** | TRBV7-9*01 | TRBJ1-2*01 | CASSADVGPVAF | **133** | | TRBV24-1*01 | TRBJ1-2*01 | CATSGDTNYGYTF | |
| **41** | TRBV28*01 | TRBJ1-1*01 | CASSLPTGGTEAFF | **134** | | TRBV28*01 | TRBJ1-6*01 | CASSFSVGESYNSPLHF | |
| **42** | TRBV5-1*01 | TRBJ2-3*01 | CASSLGSNTDTQYF | **135** | | TRBV12-3*01 | TRBJ1-1*01 | CASRATGGLNTEAFF | |
| **43** | TRBV2*01 | TRBJ1-6*01 | CASSAADHSPLHF | **136** | | TRBV7-2*01 | TRBJ2-2*01 | CASSFHSGQSNTGELFF | |
| **44** | TRBV5-1*01 | TRBJ2-7*01 | CASSLAPGEQYF | **137** | | TRBV9*01 | TRBJ1-1*01 | CASSPSVAIVGTEAFF | |
| **45** | TRBV27*01 | TRBJ2-7*01 | CASSRGQSNEQYF | **138** | | TRBV10-2*01 | TRBJ1-5*01 | CASSELKQASNQPQHF | |
| **46** | TRBV27*01 | TRBJ1-4*01 | CASSPYRVGRLYEKLFF | **139** | | TRBV6-5*01 | TRBJ2-6*01 | CASSPGQVFSGANVLTF | |
| **47** | TRBV2*01 | TRBJ2-1*01 | CASSEDPGLVNEQFF | **140** | | TRBV7-6*01 | TRBJ2-1*01 | CASSFELAGVDNEQFF | |
| **48** | TRBV27*01 | TRBJ2-1*01 | CASRNFDHNEQFF | **141** | | TRBV12-3*01 | TRBJ1-2*01 | CASSWAVADGYTF | |
| **49** | TRBV20-1*01 | TRBJ2-1*01 | CSAIDLAGGGGEQFF | **142** | | TRBV7-2*01 | TRBJ2-3*01 | CASSLASAGRSSTDTQYF | |
| **50** | TRBV6-5*01 | TRBJ2-5*01 | CASRPLAGGPETQYF | **143** | | TRBV7-2*01 | TRBJ2-6*01 | CASSPEAPHQGANVLTF | |
| **51** | TRBV15*01 | TRBJ2-4*01 | CATSRGHGLRNIQYF | **144** | | TRBV2*01 | TRBJ1-2*01 | CASSDPMLIYGYTF | |
| **52** | TRBV28*01 | TRBJ2-3*01 | CASSWDNTDTQYF | **145** | | TRBV9*01 | TRBJ2-7*01 | CASSAGQGVTYEQYF | |
| **53** | TRBV6-1*01 | TRBJ2-7*01 | CAGRSSYEQYF | **146** | | TRBV4-1*01 | TRBJ1-1*01 | CASSQAGTGLNTEAFF | |
| **54** | TRBV27*01 | TRBJ1-1*01 | CASSLGQGTEAFF | | **147** | TRBV7-9*01 | TRBJ2-1*01 | CASSLTRGRENEQFF | |
| **55** | TRBV21-1*01 | TRBJ2-7*01 | CASTF | | **148** | TRBV7-9*01 | TRBJ2-1*01 | CASSSPGQGRGYNEQFF | |
| **56** | TRBV25-1*01 | TRBJ2-7*01 | CASSVGSGGYEQYF | | **149** | TRBV28*01 | TRBJ1-2*01 | CASTLGVDGYTF | |
| **57** | TRBV27*01 | TRBJ2-7*01 | CASHGAGVNEQYF | | **150** | TRBV7-9*01 | TRBJ2-7*01 | CASSRGGSYEQYF | |
| **58** | TRBV2*01 | TRBJ2-1*01 | CASSEAGLASEQFF | | **151** | TRBV24-1*01 | TRBJ2-1*01 | CATSDRDYYNEQFF | |
| **59** | TRBV9*01 | TRBJ2-7*01 | CASSVGARRYREMPYEQYF | | **152** | TRBV9*01 | TRBJ1-3*01 | CASSVGTGVSSGNTIYF | |
| **60** | TRBV5-1*01 | TRBJ2-3*01 | CASSLESPGTSTDTQYF | | **153** | TRBV9*01 | TRBJ2-1*01 | CASSVNSNNEQFF | |
| **61** | TRBV7-6*01 | TRBJ1-3*01 | CASSLSWEGASSGNTIYF | | **154** | TRBV2*01 | TRBJ1-5*01 | CASSADFYSNQPQHF | |
| **62** | TRBV27*01 | TRBJ2-7*01 | CASGVYEQYF | **155** | | TRBV7-8*01 | TRBJ2-1*01 | CASSLGGSSYNEQFF | |
| **63** | TRBV5-1*01 | TRBJ2-2*01 | CASSGSGELFF | **156** | | TRBV7-8*01 | TRBJ2-7*01 | CASSLGQAYEQYF | |
| **64** | TRBV11-2*01 | TRBJ2-1*01 | CASSLPLGGLAGSSSYNEQFF | **157** | | TRBV5-1*01 | TRBJ1-4*01 | CASSLDLQVPTTNEKLFF | |
| **65** | TRBV5-6*01 | TRBJ2-7*01 | CASSFVGGGYEQYF | **158** | | TRBV7-7*01 | TRBJ2-1*01 | CASSLGLDAYNEQFF | |
| **66** | TRBV7-3*01 | TRBJ2-7*01 | CASSLTSYYEQYF | **159** | | TRBV12-4*01 | TRBJ1-2*01 | CASSWAVADGYTF | |
| **67** | TRBV7-6*01 | TRBJ1-1*01 | CASSLAGSYNEAFF | **160** | | TRBV4-1*01 | TRBJ2-2*01 | CASSQDAREGELFF | |
| **68** | TRBV7-9*01 | TRBJ1-5*01 | CASSLVSDSNQPQHF | **161** | | TRBV19*01 | TRBJ1-1*01 | CASSPGQGWTEAFF | |
| **69** | TRBV24-1*01 | TRBJ2-5*01 | CATSDGLEEEETQYF | **162** | | TRBV9*01 | TRBJ2-7*01 | CASSVEGTSGYEQYF | |
| **70** | TRBV2*01 | TRBJ2-2*01 | CASSEASHGELFF | **163** | | TRBV27*01 | TRBJ2-6*01 | CASSPRTGGYGANVLTF | |
| **71** | TRBV5-1*01 | TRBJ2-2*01 | CASSFGGELFF | **164** | | TRBV29-1*01 | TRBJ2-7*01 | CSASEGAGDYEQYF | |
| **72** | TRBV11-2*01 | TRBJ2-7*01 | CASSPAGRHEQYF | **165** | | TRBV4-1*01 | TRBJ2-2*01 | CASSRNSGALNTGELFF | |
| **73** | TRBV21-1*01 | TRBJ1-1*01 | CASSKGSGGTYTEAFF | **166** | | TRBV25-1*01 | TRBJ2-5*01 | CASSDLTGDETQYF | |
| **74** | TRBV27*01 | TRBJ2-1*01 | CASSLTSGSFAYNEQFF | **167** | | TRBV5-1*01 | TRBJ2-7*01 | CASSLGQGATSYEQYF | |
| **75** | TRBV10-3*01 | TRBJ2-1*01 | CAISPGTGYNEQFF | **168** | | TRBV7-8*01 | TRBJ2-7*01 | CASSLSGAYEQYF | |
| **76** | TRBV20-1*01 | TRBJ2-1*01 | CSARVGSHEQFF | **169** | | TRBV15*01 | TRBJ2-2*01 | CATSSRQLTNTGELFF | |
| **77** | TRBV10-3*01 | TRBJ2-1*01 | CAIGDEESYNEQFF | **170** | | TRBV28*01 | TRBJ2-1*01 | CASSSATTGSSYNEQFF | |
| **78** | TRBV28*01 | TRBJ1-1*01 | CASSLYTEAFF | **171** | | TRBV7-9*01 | TRBJ2-1*01 | CASSSSTPRATGNEQFF | |
| **79** | TRBV29-1*01 | TRBJ2-1*01 | CSVVANEQFF | **172** | | TRBV2*01 | TRBJ2-3*01 | CASSESPGALDTQYF | |
| **80** | TRBV7-9*01 | TRBJ1-1*01 | CASSDVRNTEAFF | **173** | | TRBV7-8*01 | TRBJ1-5*01 | CASSSMGNQPQHF | |
| **81** | TRBV20-1*01 | TRBJ2-2*01 | CSASGPAMSRELFF | **174** | | TRBV4-2*01 | TRBJ2-7*01 | CASSQDGGGSYEQYF | |
| **82** | TRBV19*01 | TRBJ2-2*01 | CVSGSLNTGELFF | **175** | | TRBV7-9*01 | TRBJ2-1*01 | CASSGLTGTGTYNEQFF | |
| **83** | TRBV18*01 | TRBJ1-5*01 | CASSPGGNQPQHF | **176** | | TRBV7-9*01 | TRBJ2-1*01 | CASSLAWTGEQFF | |
| **84** | TRBV5-4*01 | TRBJ2-1*01 | CASSWGLLNEQFF | **177** | | TRBV27*01 | TRBJ2-7*01 | CASSLGQATYEQYF | |
| **85** | TRBV9*01 | TRBJ2-7*01 | CASSVATSREQYF | **178** | | TRBV20-1*01 | TRBJ2-7*01 | CSVRALGLYEQYF | |
| **86** | TRBV12-3*01 | TRBJ2-7*01 | CASSLAGFGEQYF | **179** | | TRBV2*01 | TRBJ1-2*01 | CASSVGSRHYGYTF | |
| **87** | TRBV12-3*01 | TRBJ1-1*01 | CASSFGGTNTEAFF | **180** | | TRBV3-1*01 | TRBJ2-7*01 | CASSQDGSGPTYEQYF | |
| **88** | TRBV27*01 | TRBJ2-5*01 | CASSLSRAQETQYF | **181** | | TRBV9*01 | TRBJ2-6*01 | CASSPGQGGSGANVLTF | |
| **89** | TRBV5-1*01 | TRBJ1-1*01 | CASSLLGVGDFTNTEAFF | **182** | | TRBV12-3*01 | TRBJ2-7*01 | CASSLSYEQYF | |
| **90** | TRBV5-1*01 | TRBJ1-1*01 | CASSPWDGSNTEAFF | **183** | | TRBV6-5*01 | TRBJ1-1*01 | CASSYLTGEAFF | |
| **91** | TRBV9*01 | TRBJ2-7*01 | CASSRGLAGTYEQYF | **184** | | TRBV27*01 | TRBJ2-7*01 | CASSWGGLATSYEQYF | |
| **92** | TRBV27*01 | TRBJ1-2*01 | CASSFGPVGGIGYTF | **185** | | TRBV7-2*01 | TRBJ2-7*01 | CASSKRVGSYEQYF | |
| **93** | TRBV12-4*01 | TRBJ1-1*01 | CASSFGGTNTEAFF |  | |  |  |  |  |

| **TCRB repertoire in tumor B4 (0.1% or higher)** | | | | | | | |
| --- | --- | --- | --- | --- | --- | --- | --- |
| **No.** | **Vseg** | **Jseg** | **CDR3** | **No.** | **Vseg** | **Jseg** | **CDR3** |
| **1** | TRBV20-1*01 | TRBJ2-2*01 | CSARRGQSGYTGELFF | **87** | TRBV10-2*01 | TRBJ2-7*01 | CASRLTVGELYEQYF |
| **2** | TRBV20-1*01 | TRBJ1-2*01 | CSARDRPGDGYTF | **88** | TRBV20-1*01 | TRBJ2-1*01 | CSARDASLIGNEQFF |
| **3** | TRBV5-1*01 | TRBJ2-7*01 | CASSPGTSGFPGEQYF | **89** | TRBV5-5*01 | TRBJ2-2*01 | CASSLFGTSNTGELFF |
| **4** | TRBV28*01 | TRBJ2-3*01 | CASSLGKLLTSTDTQYF | **90** | TRBV29-1*01 | TRBJ2-7*01 | CSAERGSGTYEQYF |
| **5** | TRBV7-6*01 | TRBJ2-1*01 | CASSLLAGSYNEQFF | **91** | TRBV6-1*01 | TRBJ1-5*01 | CASSEASVDQPQHF |
| **6** | TRBV5-1*01 | TRBJ1-5*01 | CASSLNPGSQPQHF | **92** | TRBV28*01 | TRBJ2-7*01 | CASRNSSGEQYF |
| **7** | TRBV11-2*01 | TRBJ2-5*01 | CASSSYGTIGAETQYF | **93** | TRBV11-1*01 | TRBJ2-5*01 | CASSAPGLAGKETQYF |
| **8** | TRBV20-1*01 | TRBJ2-7*01 | CSAGGRGLLEQYF | **94** | TRBV7-6*01 | TRBJ2-2*01 | CASSPGAATGELFF |
| **9** | TRBV7-2*01 | TRBJ1-2*01 | CASSGQTYGYTF | **95** | TRBV2*01 | TRBJ2-7*01 | CASHGLAGTYEQYF |
| **10** | TRBV18*01 | TRBJ1-2*01 | CASSPPGPIIGYGYTF | **96** | TRBV19*01 | TRBJ2-1*01 | CATLRRGIGNEQFF |
| **11** | TRBV20-1*01 | TRBJ2-7*01 | CSARAPAGTSGSSIVSYEQYF | **97** | TRBV20-1*01 | TRBJ1-4*01 | CSAPLRAGLEATNEKLFF |
| **12** | TRBV5-1*01 | TRBJ2-7*01 | CASSSGQTYEQYF | **98** | TRBV29-1*01 | TRBJ2-5*01 | CSVANVRQETQYF |
| **13** | TRBV20-1*01 | TRBJ1-1*01 | CSARRTADTEAFF | **99** | TRBV7-2*01 | TRBJ2-1*01 | CASSTRTRRNEQFF |
| **14** | TRBV20-1*01 | TRBJ1-5*01 | CSAATWAGTGGGDQPQHF | **100** | TRBV5-5*01 | TRBJ1-1*01 | CASSWLGLGEAFF |
| **15** | TRBV3-1*01 | TRBJ2-5*01 | CASEGLRETQYF | **101** | TRBV28*01 | TRBJ2-7*01 | CASTRSYEQYF |
| **16** | TRBV20-1*01 | TRBJ1-5*01 | CSARNDRVNQPQHF | **102** | TRBV14*01 | TRBJ2-4*01 | CASSRLAEANNIQYF |
| **17** | TRBV7-6*01 | TRBJ2-7*01 | CASSLQAGGPYEQYF | **103** | TRBV7-9*01 | TRBJ2-1*01 | CASSFLGGDEQFF |
| **18** | TRBV5-1*01 | TRBJ2-5*01 | CASSQIYRGETQYF | **104** | TRBV19*01 | TRBJ1-5*01 | CASLPEGGVNQPQHF |
| **19** | TRBV5-1*01 | TRBJ2-5*01 | CASSSSGSTQYF | **105** | TRBV20-1*01 | TRBJ2-7*01 | CSARDPQRGYEQYF |
| **20** | TRBV5-1*01 | TRBJ2-1*01 | CASSWTSGAYNEQFF | **106** | TRBV29-1*01 | TRBJ2-1*01 | CSVGTSGSLLF |
| **21** | TRBV6-1*01 | TRBJ1-4*01 | CASSRQPTNEKLFF | **107** | TRBV27*01 | TRBJ1-1*01 | CASSLSVRVGGTEAFF |
| **22** | TRBV7-6*01 | TRBJ2-2*01 | CASSPMNTGELFF | **108** | TRBV5-6*01 | TRBJ2-7*01 | CASSLGTGYYEQYF |
| **23** | TRBV5-1*01 | TRBJ2-3*01 | CASSSARVGDTQYF | **109** | TRBV5-6*01 | TRBJ2-5*01 | CASSLGGGFQETQYF |
| **24** | TRBV7-6*01 | TRBJ2-7*01 | CASSLEAGGPYEQYF | **110** | TRBV5-4*01 | TRBJ2-7*01 | CASSLLHEQYF |
| **25** | TRBV7-2*01 | TRBJ1-5*01 | CASSPDRGGNQPQHF | **111** | TRBV12-4*01 | TRBJ2-3*01 | CASRPSGGGADTQYF |
| **26** | TRBV7-6*01 | TRBJ2-2*01 | CASSLQPTNTGELFF | **112** | TRBV6-1*01 | TRBJ2-5*01 | CASSGQYQETQYF |
| **27** | TRBV30*01 | TRBJ1-5*01 | CAWSVFMGTGGHQPQHF | **113** | TRBV20-1*01 | TRBJ2-1*01 | CRAGRGSYNEQFF |
| **28** | TRBV7-2*01 | TRBJ2-1*01 | CASSLEPTNEQFF | **114** | TRBV20-1*01 | TRBJ2-7*01 | CSAGTPQTYEQYF |
| **29** | TRBV27*01 | TRBJ2-6*01 | CASSYQTLDSSGANVLTF | **115** | TRBV30*01 | TRBJ1-2*01 | CAWSGGTHYGYTF |
| **30** | TRBV5-1*01 | TRBJ2-7*01 | CASNSQGGYEQYF | **116** | TRBV12-3*01 | TRBJ2-3*01 | CASRPSGGGADTQYF |
| **31** | TRBV20-1*01 | TRBJ2-3*01 | CSAIRRDGTDTQYF | **117** | TRBV5-1*01 | TRBJ2-3*01 | CASTLQGGADTQYF |
| **32** | TRBV7-2*01 | TRBJ1-1*01 | CASSLYPGRDEAFF | **118** | TRBV15*01 | TRBJ1-5*01 | CATSGRDRLNQPQHF |
| **33** | TRBV20-1*01 | TRBJ2-1*01 | CSAPPSGRAEDEQFF | **119** | TRBV10-3*01 | TRBJ2-3*01 | CAIRGGLAGEGTDTQYF |
| **34** | TRBV10-2*01 | TRBJ1-6*01 | CASSRQNSPLHF | **120** | TRBV10-3*01 | TRBJ2-3*01 | CAILGGGPTDTQYF |
| **35** | TRBV2*01 | TRBJ2-2*01 | CASSEVAPGELFF | **121** | TRBV10-3*01 | TRBJ1-1*01 | CAVSRGNTEAFF |
| **36** | TRBV3-1*01 | TRBJ2-7*01 | CASSHLNTYEQYF | **122** | TRBV7-6*01 | TRBJ2-5*01 | CASSLNGETQYF |
| **37** | TRBV5-6*01 | TRBJ2-3*01 | CASSQGGTTDTQYF | **123** | TRBV2*01 | TRBJ1-2*01 | CASSHRSGDVYGYTF |
| **38** | TRBV4-1*01 | TRBJ1-1*01 | CASREVGRGNTEAFF | **124** | TRBV27*01 | TRBJ2-1*01 | CASSPLGGAYNEQFF |
| **39** | TRBV6-1*01 | TRBJ1-1*01 | CASITAGGRNTEAFF | **125** | TRBV28*01 | TRBJ2-3*01 | CASIRDSTDTQYF |
| **40** | TRBV5-1*01 | TRBJ2-7*01 | CASSLYGIEQYF | **126** | TRBV7-2*01 | TRBJ1-2*01 | CASSLATTLAPAGYTF |
| **41** | TRBV7-6*01 | TRBJ2-5*01 | CASSLRPSNQETQYF | **127** | TRBV4-1*01 | TRBJ2-1*01 | CASSQVVASSGGGDNEQFF |
| **42** | TRBV20-1*01 | TRBJ2-5*01 | CSARGQLETQYF | **128** | TRBV20-1*01 | TRBJ2-5*01 | CSARGAGAQYF |
| **43** | TRBV20-1*01 | TRBJ2-7*01 | CSAPSQGTGNEQYF | **129** | TRBV7-9*01 | TRBJ1-5*01 | CASSLDEVQPQHF |
| **44** | TRBV5-1*01 | TRBJ2-5*01 | CASSLYPARADQGETQYF | **130** | TRBV4-1*01 | TRBJ2-1*01 | CASSWTSGEDEQFF |
| **45** | TRBV5-1*01 | TRBJ1-2*01 | CASSLETGVYYGYTF | **131** | TRBV7-2*01 | TRBJ1-6*01 | CASSFTPITSSYSPLHF |
| **46** | TRBV5-5*01 | TRBJ1-5*01 | CASSLEGRYQPQHF | **132** | TRBV2*01 | TRBJ2-3*01 | CASTPWVLAATDTQYF |
| **47** | TRBV5-1*01 | TRBJ2-1*01 | CASSPWGTGGSNEQFF | **133** | TRBV13*01 | TRBJ1-4*01 | CASSLFPSRGEKLFF |
| **48** | TRBV5-1*01 | TRBJ2-7*01 | CASSLAFGGRSYEQYF | **134** | TRBV5-1*01 | TRBJ1-2*01 | CASSHLLTGGNYGYTF |
| **49** | TRBV30*01 | TRBJ1-6*01 | CAWSPVSILNSPLHF | **135** | TRBV4-2*01 | TRBJ2-1*01 | CASSQGIYNEQFF |
| **50** | TRBV7-9*01 | TRBJ1-2*01 | CASSVGHRWGDGYTF | **136** | TRBV6-1*01 | TRBJ2-1*01 | CASSELGGNEQFF |
| **51** | TRBV27*01 | TRBJ2-7*01 | CASSPFMSSYEQYF | **137** | TRBV20-1*01 | TRBJ1-5*01 | CSARPPGTGMAPQHF |
| **52** | TRBV20-1*01 | TRBJ1-1*01 | CSQRPTGNTEAFF | **138** | TRBV15*01 | TRBJ1-5*01 | CATSSIRARGYQPQHF |
| **53** | TRBV5-1*01 | TRBJ2-3*01 | CASAPAGLNTQYF | **139** | TRBV6-5*01 | TRBJ1-1*01 | CASRLQGVNTEAFF |
| **54** | TRBV5-1*01 | TRBJ2-2*01 | CASSGGLYTGELFF | **140** | TRBV7-2*01 | TRBJ1-1*01 | CASSSRTGGVNTEAFF |
| **55** | TRBV5-1*01 | TRBJ2-7*01 | CASSLTASYEQYF | **141** | TRBV5-1*01 | TRBJ2-5*01 | CASNRQGSGETQYF |
| **56** | TRBV7-9*01 | TRBJ2-7*01 | CASTWGGGSEQYF | **142** | TRBV13*01 | TRBJ2-7*01 | CASSPPRVDYEQYF |
| **57** | TRBV10-3*01 | TRBJ2-5*01 | CAISERGQETQYF | **143** | TRBV28*01 | TRBJ2-7*01 | CASSAGPYEQYF |
| **58** | TRBV5-1*01 | TRBJ1-5*01 | CASSLDDQPQHF | **144** | TRBV20-1*01 | TRBJ1-1*01 | CSAREVVIGNTEAFF |
| **59** | TRBV5-6*01 | TRBJ2-5*01 | CASSRAPGVETQYF | **145** | TRBV27*01 | TRBJ2-1*01 | CASSLGRLTAYNEQFF |
| **60** | TRBV2*01 | TRBJ2-1*01 | CASSLDRLAYEQFF | **146** | TRBV5-6*01 | TRBJ2-7*01 | CASSLAMDRGDEQYF |
| **61** | TRBV12-4*01 | TRBJ2-6*01 | CASSSSGGAGANVLTF | **147** | TRBV5-1*01 | TRBJ1-5*01 | CASSLAPGVGQPQHF |
| **62** | TRBV4-2*01 | TRBJ2-3*01 | CASSQGVGLAGDTQYF | **148** | TRBV10-3*01 | TRBJ1-2*01 | CAISPREGPNYGYTF |
| **63** | TRBV20-1*01 | TRBJ2-3*01 | CSAGRSLAGGPAQYF | **149** | TRBV6-1*01 | TRBJ1-1*01 | CASRGWVIGTEAFF |
| **64** | TRBV20-1*01 | TRBJ2-5*01 | CRATSGSQETQYF | **150** | TRBV6-5*01 | TRBJ1-3*01 | CASRDDRSSGNTIYF |
| **65** | TRBV20-1*01 | TRBJ1-5*01 | CSGKTGEDQPQHF | **151** | TRBV27*01 | TRBJ2-7*01 | CASSLGVGSGPYEQYF |
| **66** | TRBV7-9*01 | TRBJ1-6*01 | CASRPNLGNSPLHF | **152** | TRBV7-7*01 | TRBJ2-1*01 | CASSLTSGGRNEQFF |
| **67** | TRBV20-1*01 | TRBJ2-1*01 | CSAEARTSGSGSSYNEQFF | **153** | TRBV6-1*01 | TRBJ2-1*01 | CASSEAGRRYNEQFF |
| **68** | TRBV12-3*01 | TRBJ2-6*01 | CASSSSGGAGANVLTF | **154** | TRBV20-1*01 | TRBJ2-7*01 | CSARRQEITYEQYF |
| **69** | TRBV29-1*01 | TRBJ2-2*01 | CSVTDRENTGELFF | **155** | TRBV29-1*01 | TRBJ2-1*01 | CSMRLAGVRGNEQFF |
| **70** | TRBV5-1*01 | TRBJ2-3*01 | CASRYSTDTQYF | **156** | TRBV18*01 | TRBJ2-7*01 | CASSTYSGDYEQYF |
| **71** | TRBV11-2*01 | TRBJ1-1*01 | CASSLGPFDREDTEAFF | **157** | TRBV6-6*01 | TRBJ1-2*01 | CASIGDRGEYGYTF |
| **72** | TRBV20-1*01 | TRBJ2-2*01 | CSARSLAAVGELFF | **158** | TRBV20-1*01 | TRBJ2-1*01 | CSARSPSGTSGYFF |
| **73** | TRBV7-2*01 | TRBJ2-1*01 | CASSLGLRDRAYNEQFF | **159** | TRBV7-8*01 | TRBJ2-3*01 | CASSPTQTDTQYF |
| **74** | TRBV10-3*01 | TRBJ2-5*01 | CAISDQGQGTQYF | **160** | TRBV20-1*01 | TRBJ1-1*01 | CSASLTGQEVGAFF |
| **75** | TRBV7-2*01 | TRBJ1-5*01 | CASSLAGGQPQHF | **161** | TRBV7-9*01 | TRBJ1-1*01 | CASSYTQGNTEAFF |
| **76** | TRBV6-1*01 | TRBJ1-2*01 | CASSEYPGMSSYGYTF | **162** | TRBV27*01 | TRBJ2-7*01 | CASSGLTVDEQYF |
| **77** | TRBV11-2*01 | TRBJ2-2*01 | CASSQDPHTGELFF | **163** | TRBV4-1*01 | TRBJ1-6*01 | CASSVSTGLGALHF |
| **78** | TRBV20-1*01 | TRBJ2-3*01 | CSARVTGIGTDTQYF | **164** | TRBV20-1*01 | TRBJ2-1*01 | CSARAVGVEQFF |
| **79** | TRBV5-1*01 | TRBJ2-3*01 | CASSPGQGWADTQYF | **165** | TRBV5-1*01 | TRBJ2-5*01 | CASSLYTLETQYF |
| **80** | TRBV28*01 | TRBJ2-5*01 | CASEGQVGETQYF | **166** | TRBV13*01 | TRBJ2-7*01 | CASSLRGRLTYEQYF |
| **81** | TRBV28*01 | TRBJ2-3*01 | CASSLTGEADTQYF | **167** | TRBV4-1*01 | TRBJ2-5*01 | CASSQDREQAGVQETQYF |
| **82** | TRBV10-2*01 | TRBJ1-2*01 | CASSAGGRDGYTF | **168** | TRBV7-3*01 | TRBJ2-7*01 | CASSFTGSYEQYF |
| **83** | TRBV20-1*01 | TRBJ2-3*01 | CSARDGGGSDTQYF | **169** | TRBV7-9*01 | TRBJ2-7*01 | CASSRHRGSYEQYF |
| **84** | TRBV6-1*01 | TRBJ1-4*01 | CASSSRTFSEKLFF | **170** | TRBV9*01 | TRBJ2-3*01 | CASSVGLADTDTQYF |
| **85** | TRBV5-1*01 | TRBJ1-1*01 | CASSYRGTEAFF | **171** | TRBV2*01 | TRBJ1-2*01 | CASSARDSLYGYTF |
| **86** | TRBV20-1*01 | TRBJ1-1*01 | CSARGPLAEAFF | **172** | TRBV24-1*01 | TRBJ2-1*01 | CATSDLQQGLRNEQFF |

| **TCRB repertoire in tumor B5 (0.1% or higher)** | | | | | | | | | |
| --- | --- | --- | --- | --- | --- | --- | --- | --- | --- |
| **No.** | **Vseg** | **Jseg** | **CDR3** | **No.** | **Vseg** | **Jseg** | | **CDR3** | |
| **1** | TRBV25-1*01 | TRBJ1-1*01 | CASSEYRTNTEAFF | **66** | TRBV18*01 | | TRBJ2-1*01 | | CASSLSDRETKFF |
| **2** | TRBV7-9*01 | TRBJ2-5*01 | CASSLTAGTGQETQYF | **67** | TRBV7-2*01 | | TRBJ2-2*01 | | CASSLGRGGYTGELFF |
| **3** | TRBV5-1*01 | TRBJ1-2*01 | CASSPAGIAGGYTF | **68** | TRBV2*01 | | TRBJ2-2*01 | | CASSGTTNTGELFF |
| **4** | TRBV21-1*01 | TRBJ2-7*01 | CASSKIPGQGMESEKGQYF | **69** | TRBV20-1*01 | | TRBJ2-7*01 | | CSAPTQGAREQYF |
| **5** | TRBV4-1*01 | TRBJ2-1*01 | CASSQDEGSSYNEQFF | **70** | TRBV28*01 | | TRBJ2-7*01 | | CASRIGSRGSYEQYF |
| **6** | TRBV20-1*01 | TRBJ2-3*01 | CSAESPSTDTQYF | **71** | TRBV5-1*01 | | TRBJ2-2*01 | | CASSLVLAGNTGELFF |
| **7** | TRBV20-1*01 | TRBJ2-4*01 | CSASMIGREQTKNIQYF | **72** | TRBV7-3*01 | | TRBJ2-7*01 | | CASSLTLGYEQYF |
| **8** | TRBV6-1*01 | TRBJ2-1*01 | CASSGGVSYNEQFF | **73** | TRBV20-1*01 | | TRBJ2-1*01 | | CSAATGIRSYNEQFF |
| **9** | TRBV29-1*01 | TRBJ2-3*01 | CSVDGSQETNTDTQYF | **74** | TRBV28*01 | | TRBJ1-2*01 | | CASSSLQRRGYTF |
| **10** | TRBV20-1*01 | TRBJ1-5*01 | CSAAYRSGGNQPQHF | **75** | TRBV5-1*01 | | TRBJ2-3*01 | | CASSWYPSGGGTDTQYF |
| **11** | TRBV20-1*01 | TRBJ2-3*01 | CSATRAGTRDTQYF | **76** | TRBV7-9*01 | | TRBJ1-2*01 | | CASSPTGAKGYTF |
| **12** | TRBV20-1*01 | TRBJ1-5*01 | CSARDTGLGNQPQHF | **77** | TRBV7-9*01 | | TRBJ2-1*01 | | CASTIRAGEQFF |
| **13** | TRBV7-2*01 | TRBJ2-1*01 | CASSLAAGANEQFF | **78** | TRBV14*01 | | TRBJ2-3*01 | | CASSRTGVPTQYF |
| **14** | TRBV12-4*01 | TRBJ2-1*01 | CASSFGVSYNEQFF | **79** | TRBV20-1*01 | | TRBJ2-1*01 | | CSADTDRYNEQFF |
| **15** | TRBV12-3*01 | TRBJ2-1*01 | CASSFGVSYNEQFF | **80** | TRBV5-6*01 | | TRBJ2-1*01 | | CASSLAGTEARASYNEQFF |
| **16** | TRBV30*01 | TRBJ2-1*01 | CAWSVRSQGGGNEQFF | **81** | TRBV3-1*01 | | TRBJ2-7*01 | | CASTLGASRDEQYF |
| **17** | TRBV7-2*01 | TRBJ1-3*01 | CASSYDGNTIYF | **82** | TRBV28*01 | | TRBJ2-7*01 | | CASSRQLGVLYNEQYF |
| **18** | TRBV20-1*01 | TRBJ1-1*01 | CSAKDRSTRLNTEAFF | **83** | TRBV5-1*01 | | TRBJ2-1*01 | | CASSSSPGVNEQFF |
| **19** | TRBV28*01 | TRBJ2-7*01 | CASSLPSGTYEQYF | **84** | TRBV5-1*01 | | TRBJ1-5*01 | | CASSSNPGQPQHF |
| **20** | TRBV28*01 | TRBJ2-2*01 | CASTGSGRANTGELFF | **85** | TRBV30*01 | | TRBJ2-5*01 | | CAWDLQGWETQYF |
| **21** | TRBV18*01 | TRBJ2-7*01 | CASSPQRDEQYF | **86** | TRBV7-2*01 | | TRBJ2-1*01 | | CASSLERNEQFF |
| **22** | TRBV7-2*01 | TRBJ2-7*01 | CASSSGLAYSYEQYF | **87** | TRBV20-1*01 | | TRBJ1-2*01 | | CSASPRPYTF |
| **23** | TRBV7-2*01 | TRBJ2-7*01 | CASSWGSGGYEQYF | **88** | TRBV7-6*01 | | TRBJ2-1*01 | | CASSVVGTARNEQFF |
| **24** | TRBV7-3*01 | TRBJ2-3*01 | CASSLMQTQWSTDTQYF | **89** | TRBV6-5*01 | | TRBJ2-2*01 | | CASSLAGYGELFF |
| **25** | TRBV29-1*01 | TRBJ1-4*01 | CSVVGQGLEKLFF | **90** | TRBV7-3*01 | | TRBJ2-2*01 | | CASSQDGELFF |
| **26** | TRBV7-2*01 | TRBJ1-5*01 | CASSQDGLGNQPQHF | **91** | TRBV10-3*01 | | TRBJ1-4*01 | | CATLQGFGEKLFF |
| **27** | TRBV20-1*01 | TRBJ2-7*01 | CSAGRNRESYEQYF | **92** | TRBV20-1*01 | | TRBJ2-5*01 | | CSALADQETQYF |
| **28** | TRBV12-3*01 | TRBJ1-2*01 | CASSLGTTGARGYTF | **93** | TRBV12-3*01 | | TRBJ2-5*01 | | CASSPTLGLETQYF |
| **29** | TRBV30*01 | TRBJ2-2*01 | CAWTREEIGELFF | **94** | TRBV20-1*01 | | TRBJ2-7*01 | | CSAGGQNRITEQYF |
| **30** | TRBV12-4*01 | TRBJ1-2*01 | CASSLGTTGARGYTF | **95** | TRBV5-1*01 | | TRBJ2-3*01 | | CASSLAGLDTQYF |
| **31** | TRBV29-1*01 | TRBJ2-3*01 | CSVEREGADTQYF | **96** | TRBV24-1*01 | | TRBJ2-2*01 | | CATSDPGTRTGELFF |
| **32** | TRBV7-2*01 | TRBJ1-2*01 | CASSLDFEGYTF | **97** | TRBV12-4*01 | | TRBJ2-5*01 | | CASSPTLGLETQYF |
| **33** | TRBV10-3*01 | TRBJ2-1*01 | CAISEVASPYNEQFF | **98** | TRBV7-3*01 | | TRBJ1-3*01 | | CASSYDGNTIYF |
| **34** | TRBV24-1*01 | TRBJ2-2*01 | CATSELAGVGELFF | **99** | TRBV20-1*01 | | TRBJ2-5*01 | | CSARPGGYTETQYF |
| **35** | TRBV3-1*01 | TRBJ2-2*01 | CASSQVGQGDFTGELFF | **100** | TRBV7-9*01 | | TRBJ2-5*01 | | CASSGQGAMVETQYF |
| **36** | TRBV18*01 | TRBJ1-1*01 | CASSPEGRGTEAFF | **101** | TRBV20-1*01 | | TRBJ1-1*01 | | CSASSVVGAPEAFF |
| **37** | TRBV10-2*01 | TRBJ2-7*01 | CASSESLQQRLAREQYF | **102** | TRBV7-3*01 | | TRBJ1-6*01 | | CASSLIGDSRSPLHF |
| **38** | TRBV24-1*01 | TRBJ2-2*01 | CATSDKFTGELFF | **103** | TRBV25-1*01 | | TRBJ2-4*01 | | CASSGGTRDIQYF |
| **39** | TRBV6-5*01 | TRBJ2-3*01 | CASSYSSTGGRDTQYF | **104** | TRBV5-5*01 | | TRBJ2-1*01 | | CASSLRGGLVDEQFF |
| **40** | TRBV9*01 | TRBJ2-3*01 | CASSASGTSTDTQYF | **105** | TRBV6-1*01 | | TRBJ2-1*01 | | CASKKDANNEQFF |
| **41** | TRBV15*01 | TRBJ1-1*01 | CATSRDRTGANTEAFF | **106** | TRBV6-5*01 | | TRBJ2-7*01 | | CASSEDRDYEQYF |
| **42** | TRBV7-2*01 | TRBJ2-3*01 | CASSERGGPRHTQYF | **107** | TRBV3-1*01 | | TRBJ2-5*01 | | CASSPLGDQETQYF |
| **43** | TRBV5-1*01 | TRBJ2-7*01 | CASSFMQIDEQYF | **108** | TRBV5-1*01 | | TRBJ1-1*01 | | CASSRGRGTGNTEAFF |
| **44** | TRBV24-1*01 | TRBJ1-2*01 | CATFGFGGYTF | **109** | TRBV5-1*01 | | TRBJ2-5*01 | | CASSQDQETQYF |
| **45** | TRBV20-1*01 | TRBJ1-4*01 | CSASGGTASPTNEKLFF | **110** | TRBV5-5*01 | | TRBJ2-3*01 | | CASSFRGGAGDTQYF |
| **46** | TRBV28*01 | TRBJ2-3*01 | CASSFRESHHTDTQYF | **111** | TRBV10-3*01 | | TRBJ2-1*01 | | CAISRDSYNEQFF |
| **47** | TRBV6-5*01 | TRBJ1-5*01 | CASSYSGVGQPQHF | **112** | TRBV7-2*01 | | TRBJ2-6*01 | | CASSLALGLAGAGANVLTF |
| **48** | TRBV15*01 | TRBJ2-1*01 | CATTTQTYNEQFF | **113** | TRBV3-1*01 | | TRBJ2-7*01 | | CASSEPRAPYEQYF |
| **49** | TRBV28*01 | TRBJ2-4*01 | CASSPGQGLAKNIQYF | **114** | TRBV20-1*01 | | TRBJ1-1*01 | | CSAGSGNTEAFF |
| **50** | TRBV7-2*01 | TRBJ2-7*01 | CASSLRGQGRNEQYF | **115** | TRBV12-3*01 | | TRBJ1-1*01 | | CASSFFYEAFF |
| **51** | TRBV20-1*01 | TRBJ2-1*01 | CSARDVTSGVGEQFF | **116** | TRBV20-1*01 | | TRBJ2-3*01 | | CSAPGLRTDTQYF |
| **52** | TRBV7-8*01 | TRBJ2-7*01 | CASSATGKASYEQYF | **117** | TRBV20-1*01 | | TRBJ2-7*01 | | CSARTGVRVGVYEQYF |
| **53** | TRBV10-3*01 | TRBJ2-3*01 | CATQSTDTQYF | **118** | TRBV7-8*01 | | TRBJ1-1*01 | | CASSLVGGLNTEAFF |
| **54** | TRBV6-2*01/TRBV6-3*01 | TRBJ2-7*01 | CASILTSGAYEQYF | **119** | TRBV18*01 | | TRBJ2-7*01 | | CASSPQGYEQYF |
| **55** | TRBV29-1*01 | TRBJ1-1*01 | CSVVEGASTEAFF | **120** | TRBV5-1*01 | | TRBJ1-4*01 | | CASSLGQRNEKLFF |
| **56** | TRBV20-1*01 | TRBJ2-3*01 | CSGARFRASSTDTQYF | **121** | TRBV20-1*01 | | TRBJ2-7*01 | | CSARDRARGSYEQYF |
| **57** | TRBV15*01 | TRBJ2-3*01 | CATGLSRRENTDTQYF | **122** | TRBV5-1*01 | | TRBJ2-4*01 | | CASSASGAKNIQYF |
| **58** | TRBV4-2*01 | TRBJ2-3*01 | CASSQDRTSGAYTDTQYF | **123** | TRBV9*01 | | TRBJ1-1*01 | | CASGVGPGGEAFF |
| **59** | TRBV7-9*01 | TRBJ1-5*01 | CASSPTGGINQPQHF | **124** | TRBV5-8*01 | | TRBJ2-2*01 | | CASSIRDSQNTGELFF |
| **60** | TRBV7-2*01 | TRBJ1-2*01 | CASSLGGDYGYTF | **125** | TRBV6-5*01 | | TRBJ1-6*01 | | CASSYSQGSAQNSPLHF |
| **61** | TRBV5-6*01 | TRBJ2-3*01 | CASKERGYGTDTQYF | **126** | TRBV7-9*01 | | TRBJ1-1*01 | | CASSYDVVAFF |
| **62** | TRBV29-1*01 | TRBJ1-1*01 | CSAGEADTEAFF | **127** | TRBV20-1*01 | | TRBJ2-2*01 | | CSARDLGGTNTGELFF |
| **63** | TRBV29-1*01 | TRBJ2-1*01 | CSAVGEGEGEQFF | **128** | TRBV6-5*01 | | TRBJ1-3*01 | | CASSYSLQGSSGNTIYF |
| **64** | TRBV18*01 | TRBJ2-7*01 | CASSLHSDSYEQYF | **129** | TRBV5-1*01 | | TRBJ1-1*01 | | CASSSGQGWRTAEAFF |
| **65** | TRBV6-4*01 | TRBJ2-1*01 | CASSDSTSGFSYNEQFF | **130** | TRBV20-1*01 | | TRBJ2-6*01 | | CSARQGGRSSGANVLTF |

| **TCRB repertoire in tumor B6 (0.1% or higher)** | | | | | | | |
| --- | --- | --- | --- | --- | --- | --- | --- |
| **No.** | **Vseg** | **Jseg** | **CDR3** | **No.** | **Vseg** | **Jseg** | **CDR3** |
| **1** | TRBV6-5*01 | TRBJ2-6*01 | CASTPTGSGANVLTF | **60** | TRBV11-2*01 | TRBJ1-2*01 | CASSLGVTGDNYGYTF |
| **2** | TRBV27*01 | TRBJ2-2*01 | CASSPNGGQRTGELFF | **61** | TRBV5-1*01 | TRBJ1-3*01 | CASSQTSSGNTIYF |
| **3** | TRBV9*01 | TRBJ1-1*01 | CASSVGQGNTEAFF | **62** | TRBV5-6*01 | TRBJ1-2*01 | CASSPNQGTYGYTF |
| **4** | TRBV7-9*01 | TRBJ2-1*01 | CASSSSGTSGGYNEQFF | **63** | TRBV5-1*01 | TRBJ2-5*01 | CASSSGTDPRETQYF |
| **5** | TRBV29-1*01 | TRBJ1-2*01 | CSVVPEWENGYTF | **64** | TRBV20-1*01 | TRBJ2-3*01 | CSARGRLSTDTQYF |
| **6** | TRBV7-2*01 | TRBJ1-1*01 | CASSLGQMNTEAFF | **65** | TRBV5-4*01 | TRBJ2-5*01 | CASSRVEQETQYF |
| **7** | TRBV12-5*01 | TRBJ1-6*01 | CASGTPLGEGTWNSPLHF | **66** | TRBV7-2*01 | TRBJ1-1*01 | CASSPGDTEAFF |
| **8** | TRBV29-1*01 | TRBJ2-5*01 | CSVVREWDRETQYF | **67** | TRBV20-1*01 | TRBJ1-1*01 | CSATGQGADVNTEAFF |
| **9** | TRBV4-3*01 | TRBJ1-1*01 | CASSQGAGTGGVEAFF | **68** | TRBV4-2*01 | TRBJ2-3*01 | CASSPEGTGSTDTQYF |
| **10** | TRBV29-1*01 | TRBJ2-5*01 | CSVVPEWIRETQYF | **69** | TRBV20-1*01 | TRBJ2-1*01 | CSANPSGGAYNEQFF |
| **11** | TRBV7-9*01 | TRBJ1-5*01 | CASSQVGVRDQPQHF | **70** | TRBV20-1*01 | TRBJ1-2*01 | CSARTVRANYGYTF |
| **12** | TRBV20-1*01 | TRBJ2-2*01 | CSAPRTTGELFF | **71** | TRBV20-1*01 | TRBJ1-3*01 | CSARMQASGNTIYF |
| **13** | TRBV20-1*01 | TRBJ2-1*01 | CSARAWLSEQFF | **72** | TRBV20-1*01 | TRBJ1-1*01 | CSARENQGGYTEAFF |
| **14** | TRBV12-3*01 | TRBJ2-7*01 | CASSLDSYEQYF | **73** | TRBV10-2*01 | TRBJ2-5*01 | CASTRMYQETQYF |
| **15** | TRBV6-1*01 | TRBJ1-6*01 | CASSERGRGSPLHF | **74** | TRBV5-1*01 | TRBJ1-1*01 | CASSLTEMNTEAFF |
| **16** | TRBV12-4*01 | TRBJ2-7*01 | CASSLDSYEQYF | **75** | TRBV5-1*01 | TRBJ2-7*01 | CASSLGQSYEQYF |
| **17** | TRBV5-6*01 | TRBJ2-7*01 | CASSTDTSYEQYF | **76** | TRBV7-2*01 | TRBJ2-3*01 | CASSLPGQGLLIGRDTQYF |
| **18** | TRBV7-9*01 | TRBJ1-5*01 | CASSYTGGNQPQHF | **77** | TRBV6-6*01 | TRBJ1-5*01 | CASSYGTGNQPQHF |
| **19** | TRBV27*01 | TRBJ1-2*01 | CASSFSGGNYGYTF | **78** | TRBV25-1*01 | TRBJ2-1*01 | CASSEGRGGNEQFF |
| **20** | TRBV10-3*01 | TRBJ1-5*01 | CAIGTGDSNQPQHF | **79** | TRBV20-1*01 | TRBJ2-1*01 | CSASTSGGAVDEQFF |
| **21** | TRBV9*01 | TRBJ2-5*01 | CASSVESPQGETQYF | **80** | TRBV5-1*01 | TRBJ2-1*01 | CASSLGRGLSEQFF |
| **22** | TRBV5-1*01 | TRBJ2-5*01 | CASSPGTEETQYF | **81** | TRBV11-2*01 | TRBJ2-5*01 | CASSFGGGGGETQYF |
| **23** | TRBV10-3*01 | TRBJ1-5*01 | CATSTGDSNQPQHF | **82** | TRBV29-1*01 | TRBJ1-1*01 | CSVVEGRGVTEAFF |
| **24** | TRBV5-1*01 | TRBJ2-3*01 | CASSLTGSTQYF | **83** | TRBV27*01 | TRBJ1-1*01 | CASSRTEAPEAFF |
| **25** | TRBV7-3*01 | TRBJ1-1*01 | CASSLGYGTEAFF | **84** | TRBV7-3*01 | TRBJ1-1*01 | CASSFPQAEEAFF |
| **26** | TRBV29-1*01 | TRBJ2-5*01 | CSVVREWTRETQYF | **85** | TRBV20-1*01 | TRBJ2-2*01 | CSASEGGSTGELFF |
| **27** | TRBV29-1*01 | TRBJ1-2*01 | CSVVSEWSRYGYTF | **86** | TRBV20-1*01 | TRBJ2-1*01 | CSATRGGSYNEQFF |
| **28** | TRBV7-2*01 | TRBJ1-2*01 | CASSWGQGSNYGYTF | **87** | TRBV7-9*01 | TRBJ2-1*01 | CASSRLEAGGYNEQFF |
| **29** | TRBV9*01 | TRBJ1-4*01 | CASSPSVSDSLNEKLFF | **88** | TRBV27*01 | TRBJ1-5*01 | CASSLEGYSNQPQHF |
| **30** | TRBV7-9*01 | TRBJ2-1*01 | CASSPSGSGGYNEQFF | **89** | TRBV6-1*01 | TRBJ2-7*01 | CASSDRGPSEQYF |
| **31** | TRBV5-1*01 | TRBJ2-6*01 | CASSPQGPGANVLTF | **90** | TRBV6-6*01 | TRBJ1-1*01 | CASSYRDDDTMNTEAFF |
| **32** | TRBV7-9*01 | TRBJ2-1*01 | CASSLLLAGGYNEQFF | **91** | TRBV7-2*01 | TRBJ2-3*01 | CASSLVGSGGARDTQYF |
| **33** | TRBV6-5*01 | TRBJ2-7*01 | CASSYSTLSPGTEGWEQYF | **92** | TRBV7-9*01 | TRBJ2-1*01 | CASSQGGTSRSSYNEQFF |
| **34** | TRBV7-8*01 | TRBJ2-1*01 | CASSFPISVAGEDEQFF | **93** | TRBV5-1*01 | TRBJ2-1*01 | CASSWEMGGPLDEQFF |
| **35** | TRBV7-2*01 | TRBJ2-7*01 | CASSSKQGVSYEQYF | **94** | TRBV20-1*01 | TRBJ2-1*01 | CSAREPQHLRNEQFF |
| **36** | TRBV20-1*01 | TRBJ2-2*01 | CSAPGPLGLAGELFF | **95** | TRBV30*01 | TRBJ2-7*01 | CAWRETSGASYEQYF |
| **37** | TRBV6-5*01 | TRBJ2-1*01 | CASSSDSRAYNEQFF | **96** | TRBV20-1*01 | TRBJ2-5*01 | CSAQGVETQYF |
| **38** | TRBV5-1*01 | TRBJ2-7*01 | CASSSPGGYEQYF | **97** | TRBV6-1*01 | TRBJ2-1*01 | CASSVRQGDRDEQFF |
| **39** | TRBV7-9*01 | TRBJ2-1*01 | CASSFWTSGGYNEQFF | **98** | TRBV5-1*01 | TRBJ1-6*01 | CASSWGMGSPLHF |
| **40** | TRBV7-3*01 | TRBJ2-3*01 | CASSLNPRGTDTQYF | **99** | TRBV7-9*01 | TRBJ1-1*01 | CASSSQVNTEAFF |
| **41** | TRBV4-3*01 | TRBJ2-1*01 | CASSQDELAVDEQFF | **100** | TRBV29-1*01 | TRBJ2-7*01 | CSAAITHEQYF |
| **42** | TRBV7-7*01 | TRBJ2-5*01 | CASSLSIATETQYF | **101** | TRBV20-1*01 | TRBJ1-1*01 | CSAQPRDTEAFF |
| **43** | TRBV20-1*01 | TRBJ2-1*01 | CSARDMAGETYNEQFF | **102** | TRBV9*01 | TRBJ1-6*01 | CASSAGGQGSSPLHF |
| **44** | TRBV20-1*01 | TRBJ1-1*01 | CSARGRGMNTEAFF | **103** | TRBV20-1*01 | TRBJ1-5*01 | CSARESNQPQHF |
| **45** | TRBV29-1*01 | TRBJ1-1*01 | CSAGTGEAFF | **104** | TRBV27*01 | TRBJ1-4*01 | CASSMNWGDEKLFF |
| **46** | TRBV5-1*01 | TRBJ1-1*01 | CASSERVGNTEAFF | **105** | TRBV4-1*01 | TRBJ1-1*01 | CASSQGGAYTEAFF |
| **47** | TRBV10-3*01 | TRBJ2-7*01 | CAISGGGGGEQYF | **106** | TRBV5-4*01 | TRBJ1-4*01 | CASSRSAGDAEKLFF |
| **48** | TRBV7-7*01 | TRBJ2-1*01 | CASSSTIQDEEQFF | **107** | TRBV7-6*01 | TRBJ2-1*01 | CASSLNQDGEQFF |
| **49** | TRBV7-6*01 | TRBJ1-1*01 | CASSLRTGEAFF | **108** | TRBV5-1*01 | TRBJ2-1*01 | CASSLEGGRVDEQFF |
| **50** | TRBV6-2*01/TRBV6-3*01 | TRBJ2-7*01 | CASSYEGIGEQYF | **109** | TRBV20-1*01 | TRBJ2-1*01 | CSARDGRGQFF |
| **51** | TRBV20-1*01 | TRBJ2-3*01 | CSAPSGGALDTQYF | **110** | TRBV20-1*01 | TRBJ2-1*01 | CSARGSGGAIYNEQFF |
| **52** | TRBV13*01 | TRBJ1-1*01 | CASSPQHGQTEAFF | **111** | TRBV5-6*01 | TRBJ1-2*01 | CASSLPRTGPYGYTF |
| **53** | TRBV7-9*01 | TRBJ1-3*01 | CASSYGQSSGNTIYF | **112** | TRBV5-1*01 | TRBJ2-7*01 | CASSPGDRLAYEQYF |
| **54** | TRBV20-1*01 | TRBJ2-7*01 | CSAQRSSSYEQYF | **113** | TRBV4-3*01 | TRBJ1-6*01 | CASSQQASESPLHF |
| **55** | TRBV5-1*01 | TRBJ2-7*01 | CASSPEGSHYEQYF | **114** | TRBV9*01 | TRBJ1-1*01 | CASSLARGREGTEAFF |
| **56** | TRBV18*01 | TRBJ1-2*01 | CASSPDRGIFYGYTF | **115** | TRBV5-1*01 | TRBJ2-1*01 | CASSLEGDEQFF |
| **57** | TRBV5-1*01 | TRBJ1-2*01 | CASSSKGEGESYTF | **116** | TRBV20-1*01 | TRBJ1-5*01 | CSARNPGQGAGQPQHF |
| **58** | TRBV7-2*01 | TRBJ1-1*01 | CASRAGTEAFF | **117** | TRBV7-2*01 | TRBJ2-3*01 | CASSLGPAGGGAGDTQYF |
| **59** | TRBV7-6*01 | TRBJ1-4*01 | CASSLNYDGGRHEKLFF | **118** | TRBV7-6*01 | TRBJ2-1*01 | CASSLDIQTEEQFF |

| **TCRB repertoire in tumor B7 (0.1% or higher)** | | | | | | | |
| --- | --- | --- | --- | --- | --- | --- | --- |
| **No.** | **Vseg** | **Jseg** | **CDR3** | **No.** | **Vseg** | **Jseg** | **CDR3** |
| **1** | TRBV7-2*01 | TRBJ2-5*01 | CASSRLTGGVQETQYF | **68** | TRBV7-6*01 | TRBJ2-3*01 | CASSLAGGPSDTQYF |
| **2** | TRBV7-2*01 | TRBJ2-1*01 | CASSLVQGAMSYNEQFF | **69** | TRBV6-2*01/TRBV6-3*01 | TRBJ2-1*01 | CASRYSPRGGSSYNEQFF |
| **3** | TRBV7-9*01 | TRBJ2-1*01 | CASSPGGSRYNEQFF | **70** | TRBV7-8*01 | TRBJ2-2*01 | CASSLGGGYGTGELFF |
| **4** | TRBV11-2*01 | TRBJ1-6*01 | CASSYRGGNSPLHF | **71** | TRBV7-7*01 | TRBJ2-1*01 | CASSLAGANEQFF |
| **5** | TRBV7-2*01 | TRBJ2-7*01 | CASSLGQGASYEQYF | **72** | TRBV2*01 | TRBJ2-7*01 | CASSAGQVSYEQYF |
| **6** | TRBV7-6*01 | TRBJ1-5*01 | CATGTGGSQPQHF | **73** | TRBV5-1*01 | TRBJ2-1*01 | CASSATSGSNEQFF |
| **7** | TRBV7-2*01 | TRBJ2-7*01 | CASSLSRQTSYEQYF | **74** | TRBV24-1*01 | TRBJ2-2*01 | CATSARTSGANTGELFF |
| **8** | TRBV7-9*01 | TRBJ2-7*01 | CASSLLGDYEQYF | **75** | TRBV4-1*01 | TRBJ1-4*01 | CASSQAWPATNEKLFF |
| **9** | TRBV11-3*01 | TRBJ2-7*01 | CASSPRRGYEQYF | **76** | TRBV6-5*01 | TRBJ1-2*01 | CASSYTPRGTSHGYTF |
| **10** | TRBV19*01 | TRBJ2-3*01 | CASNSGSHTDTQYF | **77** | TRBV7-2*01 | TRBJ2-7*01 | CASSYRDREYEQYF |
| **11** | TRBV5-5*01 | TRBJ2-5*01 | CASSLRGGQETQYF | **78** | TRBV29-1*01 | TRBJ1-1*01 | CSVEWLEGGKLAFF |
| **12** | TRBV10-3*01 | TRBJ1-2*01 | CAISEQGGYSGYTF | **79** | TRBV27*01 | TRBJ1-2*01 | CASSPRGGWYVDYGYTF |
| **13** | TRBV11-2*01 | TRBJ2-7*01 | CASSLRLAGGPFSYEQYF | **80** | TRBV2*01 | TRBJ2-5*01 | CASSSDRDQAQYF |
| **14** | TRBV5-1*01 | TRBJ2-1*01 | CASRSGTSGREQFF | **81** | TRBV29-1*01 | TRBJ2-2*01 | CSVGQGATGELFF |
| **15** | TRBV20-1*01 | TRBJ2-7*01 | CSARAQSAGIAYEQYF | **82** | TRBV7-9*01 | TRBJ2-1*01 | CASSPPGSYNEQFF |
| **16** | TRBV20-1*01 | TRBJ2-7*01 | CSARASGGQGSYEQYF | **83** | TRBV7-9*01 | TRBJ2-7*01 | CASSPRDRVWLGEQYF |
| **17** | TRBV27*01 | TRBJ2-3*01 | CASRGGWDRNPSTDTQYF | **84** | TRBV18*01 | TRBJ2-3*01 | CASSPSRSRTKSTDTQYF |
| **18** | TRBV20-1*01 | TRBJ2-1*01 | CSARDLSGGWTSGSGYNEQFF | **85** | TRBV10-2*01 | TRBJ1-2*01 | CASSAEGRGYTF |
| **19** | TRBV27*01 | TRBJ2-5*01 | CASRGQEETQYF | **86** | TRBV18*01 | TRBJ2-2*01 | CASSPRRSGSGGELFF |
| **20** | TRBV6-6*01 | TRBJ2-7*01 | CASSYPLGSGESGVRAYEQYF | **87** | TRBV5-1*01 | TRBJ2-1*01 | CASSLETARVDEQFF |
| **21** | TRBV5-1*01 | TRBJ2-5*01 | CASSFSWTSGMTQYF | **88** | TRBV5-1*01 | TRBJ1-1*01 | CASSLGASAEAFF |
| **22** | TRBV5-1*01 | TRBJ2-7*01 | CASSSLDKAYEQYF | **89** | TRBV20-1*01 | TRBJ1-2*01 | CSARNQIYRDGYTF |
| **23** | TRBV5-1*01 | TRBJ2-7*01 | CASSWDKTYEQYF | **90** | TRBV5-6*01 | TRBJ2-2*01 | CASSLVQRNTGELFF |
| **24** | TRBV7-9*01 | TRBJ2-1*01 | CASSLGGSRYNEQFF | **91** | TRBV15*01 | TRBJ1-2*01 | CATSREGRQGYTF |
| **25** | TRBV11-3*01 | TRBJ2-7*01 | CASSLRGNYEQYF | **92** | TRBV20-1*01 | TRBJ1-2*01 | CSARGGQGNYGYTF |
| **26** | TRBV18*01 | TRBJ2-7*01 | CASSPHRGLDYEQYF | **93** | TRBV27*01 | TRBJ2-1*01 | CASRGWSSYNEQFF |
| **27** | TRBV29-1*01 | TRBJ1-1*01 | CSVEPRTGINTEAFF | **94** | TRBV28*01 | TRBJ1-5*01 | CASSVRPGEQPQHF |
| **28** | TRBV5-1*01 | TRBJ2-6*01 | CASRPGTAESGANVLTF | **95** | TRBV10-3*01 | TRBJ2-1*01 | CAISEGRSAYNEQFF |
| **29** | TRBV20-1*01 | TRBJ2-1*01 | CSARDLTSGRVEQFF | **96** | TRBV12-3*01 | TRBJ2-5*01 | CASSLAGPSETQYF |
| **30** | TRBV5-4*01 | TRBJ2-7*01 | CASSYPGTGVADQTF | **97** | TRBV6-5*01 | TRBJ1-1*01 | CASSYDRGNTEAFF |
| **31** | TRBV10-3*01 | TRBJ1-5*01 | CAGTTGGNSNQPQHF | **98** | TRBV20-1*01 | TRBJ2-7*01 | CSATRFLSYEQYF |
| **32** | TRBV7-9*01 | TRBJ2-1*01 | CASSKAGTYNEQFF | **99** | TRBV7-9*01 | TRBJ1-1*01 | CASSRNRGWNTEAFF |
| **33** | TRBV7-6*01 | TRBJ2-5*01 | CASSLNGALGNQETQYF | **100** | TRBV7-8*01 | TRBJ2-5*01 | CASSDPNEAGEETQYF |
| **34** | TRBV5-1*01 | TRBJ2-6*01 | CASSQASGANVLTF | **101** | TRBV12-4*01 | TRBJ2-5*01 | CASSLAGPSETQYF |
| **35** | TRBV29-1*01 | TRBJ2-7*01 | CSVASGTGTLYEQYF | **102** | TRBV18*01 | TRBJ2-6*01 | CASSTSGGSGANVLTF |
| **36** | TRBV20-1*01 | TRBJ2-3*01 | CSALPTSRLDTDTQYF | **103** | TRBV7-9*01 | TRBJ2-1*01 | CASSLRSGSRGNEQFF |
| **37** | TRBV15*01 | TRBJ2-6*01 | CATSRVGLSGANVLTF | **104** | TRBV7-6*01 | TRBJ2-1*01 | CASSPGHAILWGNEQFF |
| **38** | TRBV18*01 | TRBJ1-4*01 | CASSPGQGAKNEKLFF | **105** | TRBV29-1*01 | TRBJ1-4*01 | CSVTGTGLEKLFF |
| **39** | TRBV11-2*01 | TRBJ2-1*01 | CASNTGLAASYNEQFF | **106** | TRBV7-2*01 | TRBJ1-6*01 | CASTLGSNSPLHF |
| **40** | TRBV20-1*01 | TRBJ2-1*01 | CSAGTRTSGRARTTYNEQFF | **107** | TRBV20-1*01 | TRBJ1-3*01 | CSARAARTGELTTRGNTIYF |
| **41** | TRBV20-1*01 | TRBJ2-5*01 | CSARDRLGETQYF | **108** | TRBV7-9*01 | TRBJ2-1*01 | CASSSPGTSLTTEFF |
| **42** | TRBV5-5*01 | TRBJ1-2*01 | CASSLEFRGDGYTF | **109** | TRBV11-2*01 | TRBJ2-7*01 | CASDSGTRGEQYF |
| **43** | TRBV7-8*01 | TRBJ1-2*01 | CASSLVGTGDGYTF | **110** | TRBV15*01 | TRBJ2-7*01 | CATSRAGTGGRYEQYF |
| **44** | TRBV5-1*01 | TRBJ2-1*01 | CASSSRTSGTYEQFF | **111** | TRBV25-1*01 | TRBJ2-7*01 | CASSGVGSYEQYF |
| **45** | TRBV10-2*01 | TRBJ1-3*01 | CASSRGPDGNTIYF | **112** | TRBV29-1*01 | TRBJ2-5*01 | CSVVGQGQRRETQYF |
| **46** | TRBV5-6*01 | TRBJ2-7*01 | CASSLFTGARPSREQYF | **113** | TRBV12-3*01 | TRBJ2-7*01 | CASGSGSQGHTYEQYF |
| **47** | TRBV15*01 | TRBJ1-2*01 | CATSRDQTDYGYTF | **114** | TRBV20-1*01 | TRBJ1-4*01 | CSASSGANEKLFF |
| **48** | TRBV6-1*01 | TRBJ2-7*01 | CASTGFGTGDHEQYF | **115** | TRBV12-4*01 | TRBJ1-1*01 | CASSPGPRNTEAFF |
| **49** | TRBV5-1*01 | TRBJ1-1*01 | CASSLSGGNTEAFF | **116** | TRBV7-2*01 | TRBJ2-3*01 | CASRPQGGLADTQYF |
| **50** | TRBV20-1*01 | TRBJ2-1*01 | CRAGAAYNEQFF | **117** | TRBV10-2*01 | TRBJ2-7*01 | CASSPEGREQYF |
| **51** | TRBV5-4*01 | TRBJ2-1*01 | CASSKSGNSYNEQFF | **118** | TRBV12-3*01 | TRBJ1-1*01 | CASSPGPRNTEAFF |
| **52** | TRBV5-1*01 | TRBJ2-1*01 | CASSSTGYNEQFF | **119** | TRBV14*01 | TRBJ1-1*01 | CASSQYWGTEAFF |
| **53** | TRBV5-1*01 | TRBJ2-7*01 | CASSTGLAGGPLRGEQYF | **120** | TRBV20-1*01 | TRBJ2-1*01 | CSAAHPEQFF |
| **54** | TRBV10-3*01 | TRBJ1-1*01 | CAISEAHGGGYEAFF | **121** | TRBV13*01 | TRBJ2-1*01 | CASSLIQGYNEQFF |
| **55** | TRBV7-2*01 | TRBJ1-3*01 | CASSSAPGRGTNTIYF | **122** | TRBV12-4*01 | TRBJ2-7*01 | CASGSGSQGHTYEQYF |
| **56** | TRBV12-4*01 | TRBJ2-7*01 | CASSTRTGRYEQYF | **123** | TRBV28*01 | TRBJ1-5*01 | CASRRGSEPQHF |
| **57** | TRBV5-5*01 | TRBJ2-1*01 | CASSPRSRDNEQFF | **124** | TRBV7-9*01 | TRBJ1-5*01 | CASSLSDGPSPQHF |
| **58** | TRBV27*01 | TRBJ2-7*01 | CASSLSGQPYEQYF | **125** | TRBV20-1*01 | TRBJ1-1*01 | CSAREHLRSTEAFF |
| **59** | TRBV10-3*01 | TRBJ1-1*01 | CAISEPGQGNTEAFF | **126** | TRBV7-9*01 | TRBJ2-7*01 | CASSLGTSFYSYEQYF |
| **60** | TRBV4-3*01 | TRBJ1-5*01 | CASSQEVRETQPQHF | **127** | TRBV7-8*01 | TRBJ1-5*01 | CASGLSWDQPQHF |
| **61** | TRBV12-3*01 | TRBJ2-7*01 | CASSTRTGRYEQYF | **128** | TRBV18*01 | TRBJ1-1*01 | CASSPGLNTEAFF |
| **62** | TRBV6-1*01 | TRBJ2-1*01 | CASTLIQAGGWNEQFF | **129** | TRBV4-1*01 | TRBJ2-1*01 | CASSQEIRAVNEQFF |
| **63** | TRBV5-1*01 | TRBJ2-3*01 | CASSFEGVGTDTQYF | **130** | TRBV11-2*01 | TRBJ2-2*01 | CASSLSDTGELFF |
| **64** | TRBV20-1*01 | TRBJ2-1*01 | CSALGLAGGDEQFF | **131** | TRBV5-4*01 | TRBJ2-4*01 | CASSRTSGENIQYF |
| **65** | TRBV5-5*01 | TRBJ1-3*01 | CASSLVQAGNTIYF | **132** | TRBV12-3*01 | TRBJ2-2*01 | CASTDTGRGELFF |
| **66** | TRBV4-2*01 | TRBJ2-4*01 | CASSPLGSGIAKNIQYF | **133** | TRBV15*01 | TRBJ2-1*01 | CATSRDVRGGNEQFF |
| **67** | TRBV11-2*01 | TRBJ2-7*01 | CASSLWDRGTSGSHEQYF |  |  |  |  |

| **TCRB repertoire in tumor B8 (0.1% or higher)** | | | | | | | | |  |  |
| --- | --- | --- | --- | --- | --- | --- | --- | --- | --- | --- |
| **No.** | **Vseg** | **Jseg** | **CDR3** | **No.** | | **Vseg** | **Jseg** | **CDR3** |  |  |
| **1** | TRBV11-2*01 | TRBJ2-1*01 | CASSLEFGGSYNEQFF | **66** | TRBV20-1*01 | | TRBJ1-1*01 | CSARGPTGGNTEAFF | | |
| **2** | TRBV28*01 | TRBJ2-7*01 | CASSTQRYEQYF | **67** | TRBV12-3*01 | | TRBJ2-1*01 | CASRLGNEQFF | | |
| **3** | TRBV27*01 | TRBJ2-1*01 | CASTRSGYNEQFF | **68** | TRBV16*01 | | TRBJ1-5*01 | CASSQQGAGNQPQHF | | |
| **4** | TRBV28*01 | TRBJ1-5*01 | CATDGTGEQWQPQHF | **69** | TRBV5-1*01 | | TRBJ2-1*01 | CASSPWTAGGHRSEQFF | | |
| **5** | TRBV20-1*01 | TRBJ2-1*01 | CSAREPPYNEQFF | **70** | TRBV5-1*01 | | TRBJ1-1*01 | CASSLVAQGDTEAFF | | |
| **6** | TRBV6-6*01 | TRBJ2-5*01 | CASSRTQGETQYF | **71** | TRBV20-1*01 | | TRBJ2-1*01 | CSARGQNNEQFF | | |
| **7** | TRBV29-1*01 | TRBJ2-1*01 | CSVEKGVSYNEQFF | **72** | TRBV27*01 | | TRBJ2-1*01 | CASSLYRATPYNEQFF | | |
| **8** | TRBV20-1*01 | TRBJ2-7*01 | CSARPDRGQEQYF | **73** | TRBV24-1*01 | | TRBJ1-2*01 | CATSASDSYGYTF | | |
| **9** | TRBV20-1*01 | TRBJ1-1*01 | CSAHLQGISTEAFF | **74** | TRBV28*01 | | TRBJ1-1*01 | CASSFYRGNTEAFF | | |
| **10** | TRBV6-6*01 | TRBJ1-2*01 | CASSYSPGARDGYTF | **75** | TRBV5-1*01 | | TRBJ2-1*01 | CASSRGLAGTPRGEQFF | | |
| **11** | TRBV7-6*01 | TRBJ2-7*01 | CASSESSGNEQYF | **76** | TRBV7-2*01 | | TRBJ2-1*01 | CASSADTYNEQFF | | |
| **12** | TRBV6-6*01 | TRBJ1-1*01 | CASSKTRGEEAFF | **77** | TRBV20-1*01 | | TRBJ1-1*01 | CSAHRGQSKAFF | | |
| **13** | TRBV4-2*01 | TRBJ2-2*01 | CASKTGTSETGELFF | **78** | TRBV7-2*01 | | TRBJ2-7*01 | CASSPRDRALGEQYF | | |
| **14** | TRBV20-1*01 | TRBJ2-3*01 | CSATAGATSTDTQYF | **79** | TRBV12-3*01 | | TRBJ1-5*01 | CASSTSGQGDQPQHF | | |
| **15** | TRBV6-6*01 | TRBJ2-1*01 | CASSIYGPSYNEQFF | **80** | TRBV12-4*01 | | TRBJ1-5*01 | CASSTSGQGDQPQHF | | |
| **16** | TRBV28*01 | TRBJ2-1*01 | CASSYSGGYNEQFF | **81** | TRBV24-1*01 | | TRBJ2-3*01 | CATSDSGLSDTQYF | | |
| **17** | TRBV20-1*01 | TRBJ1-2*01 | CSAHGGGGDGYTF | **82** | TRBV10-2*01 | | TRBJ1-1*01 | CASSGDGMNTEAFF | | |
| **18** | TRBV28*01 | TRBJ2-7*01 | CASTWDRAGAYEQYF | **83** | TRBV20-1*01 | | TRBJ1-1*01 | CSACWTGWNTEAFF | | |
| **19** | TRBV7-2*01 | TRBJ1-5*01 | CASSLSGKGNQPQHF | **84** | TRBV15*01 | | TRBJ2-3*01 | CATTLDPFPEDTQYF | | |
| **20** | TRBV6-6*01 | TRBJ2-1*01 | CASSWVHNEQFF | **85** | TRBV7-9*01 | | TRBJ2-7*01 | CASSPRDRAYEQYF | | |
| **21** | TRBV7-8*01 | TRBJ2-1*01 | CASSSGHYNEQFF | **86** | TRBV28*01 | | TRBJ2-3*01 | CASSHRESGTDTQYF | | |
| **22** | TRBV5-1*01 | TRBJ1-1*01 | CASSSQGAASEAFF | **87** | TRBV6-5*01 | | TRBJ2-7*01 | CASSYRSGAPVPHF | | |
| **23** | TRBV7-6*01 | TRBJ1-1*01 | CASSFPYGEAFF | **88** | TRBV27*01 | | TRBJ2-5*01 | CASRRQGWETQYF | | |
| **24** | TRBV20-1*01 | TRBJ2-3*01 | CSASWREGQDTQYF | **89** | TRBV20-1*01 | | TRBJ2-5*01 | CSAQTGAVQETQYF | | |
| **25** | TRBV20-1*01 | TRBJ1-2*01 | CSARSQGGGAEGYTF | **90** | TRBV20-1*01 | | TRBJ2-3*01 | CSAVTPGRAQYF | | |
| **26** | TRBV7-9*01 | TRBJ1-4*01 | CASSFSWGAGEKLFF | **91** | TRBV12-3*01 | | TRBJ2-7*01 | CASSPRAGQGADEQYF | | |
| **27** | TRBV20-1*01 | TRBJ1-6*01 | CSARADSSYNSPLHF | **92** | TRBV20-1*01 | | TRBJ2-7*01 | CSANGQGTYEQYF | | |
| **28** | TRBV5-1*01 | TRBJ1-4*01 | CASSNLGTARGKLFF | **93** | TRBV4-2*01 | | TRBJ1-1*01 | CASSQGVGLRNTEAFF | | |
| **29** | TRBV20-1*01 | TRBJ1-1*01 | CSAIAGQERIEAFF | **94** | TRBV7-6*01 | | TRBJ2-2*01 | CASSLGSDRAGELFF | | |
| **30** | TRBV20-1*01 | TRBJ2-7*01 | CSALLTGGIYEQYF | **95** | TRBV29-1*01 | | TRBJ1-1*01 | CSVGHSNTEAFF | | |
| **31** | TRBV20-1*01 | TRBJ1-1*01 | CSLWGTANTEAFF | **96** | TRBV2*01 | | TRBJ2-2*01 | CASGAGLAGPTGELFF | | |
| **32** | TRBV6-6*01 | TRBJ2-7*01 | CASSYTAYEQYF | **97** | TRBV11-2*01 | | TRBJ2-2*01 | CASSLDVSNTGELFF | | |
| **33** | TRBV5-1*01 | TRBJ1-5*01 | CASSLEGGNQPQHF | **98** | TRBV3-1*01 | | TRBJ1-5*01 | CASSQGTGSYKTQHF | | |
| **34** | TRBV5-1*01 | TRBJ1-1*01 | CASSFPANTEAFF | **99** | TRBV24-1*01 | | TRBJ2-7*01 | CATSESGQGTSYEQYF | | |
| **35** | TRBV7-2*01 | TRBJ1-1*01 | CASSFRGEAFF | **100** | TRBV27*01 | | TRBJ1-2*01 | CASSPTLGVGYGYTF | | |
| **36** | TRBV29-1*01 | TRBJ2-3*01 | CSVGKRSTDTQYF | **101** | TRBV28*01 | | TRBJ2-1*01 | CASKRAGGYNEQFF | | |
| **37** | TRBV25-1*01 | TRBJ2-3*01 | CASSVVLGDTQYF | **102** | TRBV28*01 | | TRBJ2-2*01 | CASRRIQGFGGELFF | | |
| **38** | TRBV5-1*01 | TRBJ2-5*01 | CASSPGTGYQETQYF | **103** | TRBV5-1*01 | | TRBJ2-2*01 | CASSQGLAGKNTGELFF | | |
| **39** | TRBV9*01 | TRBJ2-3*01 | CASSVGGQGSTDTQYF | **104** | TRBV7-2*01 | | TRBJ2-2*01 | CASSPTSGNTGELFF | | |
| **40** | TRBV4-2*01 | TRBJ2-7*01 | CASSRGSYEQYF | **105** | TRBV5-1*01 | | TRBJ1-1*01 | CASSWDGVTEAFF | | |
| **41** | TRBV5-1*01 | TRBJ2-7*01 | CASSLSAGLIPYEQYF | **106** | TRBV5-1*01 | | TRBJ2-5*01 | CASSFSGEETQYF | | |
| **42** | TRBV28*01 | TRBJ1-5*01 | CASQIGDHGAQHF | **107** | TRBV5-1*01 | | TRBJ1-4*01 | CASSPGTGDEKLFF | | |
| **43** | TRBV20-1*01 | TRBJ2-1*01 | CSASLARSYNEQFF | **108** | TRBV27*01 | | TRBJ1-6*01 | CASKTGRDSPLHF | | |
| **44** | TRBV5-1*01 | TRBJ2-3*01 | CASSGSYLSTDTQYF | **109** | TRBV20-1*01 | | TRBJ2-1*01 | CSVGRPSSYNEQFF | | |
| **45** | TRBV5-1*01 | TRBJ1-1*01 | CASKTGTDNTEAFF | **110** | TRBV12-4*01 | | TRBJ2-7*01 | CASSPRAGQGADEQYF | | |
| **46** | TRBV20-1*01 | TRBJ1-5*01 | CSARTVEIRAESNQPQHF | **111** | TRBV5-5*01 | | TRBJ1-2*01 | CASSLEWAAGYYYGYTF | | |
| **47** | TRBV7-2*01 | TRBJ2-7*01 | CASSLAQGPDEQYF | **112** | TRBV5-1*01 | | TRBJ1-6*01 | CASSHGQGASGPLHF | | |
| **48** | TRBV29-1*01 | TRBJ1-1*01 | CSSLGGGFNTEAFF | **113** | TRBV7-2*01 | | TRBJ2-7*01 | CASSSSSGYEQYF | | |
| **49** | TRBV18*01 | TRBJ1-4*01 | CASSPRGNEKLFF | **114** | TRBV7-2*01 | | TRBJ1-5*01 | CASSSTGTGNQPQHF | | |
| **50** | TRBV20-1*01 | TRBJ1-5*01 | CSARERPGQPQHF | **115** | TRBV4-2*01 | | TRBJ1-4*01 | CASSQSHGAVFF | | |
| **51** | TRBV7-2*01 | TRBJ1-2*01 | CASSPSTGGGYTF | **116** | TRBV28*01 | | TRBJ1-2*01 | CASSLDRGQGYTF | | |
| **52** | TRBV29-1*01 | TRBJ1-2*01 | CSVELGVGYTF | **117** | TRBV13*01 | | TRBJ2-3*01 | CASSHRAAGVWADTQYF | | |
| **53** | TRBV12-4*01 | TRBJ1-3*01 | CASSLMAAGNTIYF | **118** | TRBV5-6*01 | | TRBJ2-7*01 | CASSLGAGGTYEQYF | | |
| **54** | TRBV5-1*01 | TRBJ2-3*01 | CASSLDGGTLVDTQYF | **119** | TRBV5-1*01 | | TRBJ2-7*01 | CASSKSPGLAGGLQGEQYF | | |
| **55** | TRBV24-1*01 | TRBJ2-3*01 | CATSHPGGSTDTQYF | **120** | TRBV20-1*01 | | TRBJ2-7*01 | CSARGLSYEQYF | | |
| **56** | TRBV7-8*01 | TRBJ1-1*01 | CASSGRAGGNTEAFF | **121** | TRBV12-4*01 | | TRBJ2-5*01 | CASSQTSGGRETQYF | | |
| **57** | TRBV5-1*01 | TRBJ1-1*01 | CASSSSGQGSPEAFF | **122** | TRBV10-3*01 | | TRBJ2-7*01 | CATREDSYEQYF | | |
| **58** | TRBV12-3*01 | TRBJ1-3*01 | CASSLMAAGNTIYF | **123** | TRBV11-2*01 | | TRBJ2-1*01 | CASSLEF | |  |
| **59** | TRBV7-9*01 | TRBJ2-1*01 | CASSFDGRGYNEQFF | **124** | TRBV29-1*01 | | TRBJ1-2*01 | CSVEKGQGGGYGYTF | | |
| **60** | TRBV28*01 | TRBJ2-4*01 | CASSFDRSIQYF | **125** | TRBV5-1*01 | | TRBJ1-2*01 | CASSFPGTGAGYTF | | |
| **61** | TRBV20-1*01 | TRBJ1-6*01 | CSAKGRMARNSPLHF | **126** | TRBV20-1*01 | | TRBJ2-3*01 | CSVWTSNRDTQYF | | |
| **62** | TRBV20-1*01 | TRBJ1-2*01 | CSATQGQGPRGYTF | **127** | TRBV5-1*01 | | TRBJ2-5*01 | CASSLPGAGETQYF | | |
| **63** | TRBV28*01 | TRBJ2-7*01 | CASSSWTGKRQYF | **128** | TRBV12-3*01 | | TRBJ2-5*01 | CASSQTSGGRETQYF | | |
| **64** | TRBV20-1*01 | TRBJ1-4*01 | CSASRTGGFGELFF | **129** | TRBV6-1*01 | | TRBJ2-1*01 | CASSHQANEQFF | | |
| **65** | TRBV7-9*01 | TRBJ2-1*01 | CASSVTGTPYNEQFF |  |  | |  |  | |  |
